# AMP1 and CYP78A5/7 act through a common pathway to govern cell fate maintenance in *Arabidopsis thaliana*

**DOI:** 10.1371/journal.pgen.1009043

**Published:** 2020-09-22

**Authors:** Olena Poretska, Saiqi Yang, Delphine Pitorre, Brigitte Poppenberger, Tobias Sieberer

**Affiliations:** 1 Research Unit Plant Growth Regulation, TUM School of Life Sciences Weihenstephan, Technical University of Munich, Freising, Germany; 2 Department for Microbiology, Immunobiology and Genetics, Max F. Perutz Laboratories, University of Vienna, Vienna, Austria; 3 Biotechnology of Horticultural Crops, TUM School of Life Sciences Weihenstephan, Technical University of Munich, Freising, Germany; "USDA-ARS Pacific West Area", UNITED STATES

## Abstract

Higher plants can continuously form new organs by the sustained activity of pluripotent stem cells. These stem cells are embedded in meristems, where they produce descendants, which undergo cell proliferation and differentiation programs in a spatiotemporally-controlled manner. Under certain conditions, pluripotency can be reestablished in descending cells and this reversion in cell fate appears to be actively suppressed by the existing stem cell pool. Mutation of the putative carboxypeptidase ALTERED MERISTEM PROGRAM1 (AMP1) in Arabidopsis causes defects in the suppression of pluripotency in cells normally programmed for differentiation, giving rise to unique hypertrophic phenotypes during embryogenesis as well as in the shoot apical meristem. A role of AMP1 in the miRNA-dependent control of translation has recently been established, however, how this activity is connected to its developmental functions is not resolved. Here we identify members of the cytochrome P450 clade CYP78A to act in parallel with AMP1 to control cell fate in Arabidopsis. Mutation of CYP78A5 and its close homolog CYP78A7 in a *cyp78a5*,*7* double mutant caused suspensor-to-embryo conversion and ectopic stem cell pool formation in the shoot meristem, phenotypes characteristic for *amp1*. The tissues affected in the mutants showed pronounced expression levels of *AMP1* and *CYP78A5* in wild type. A comparison of mutant transcriptomic responses revealed an intriguing degree of overlap and highlighted alterations in protein lipidation processes. Moreover, we also found elevated protein levels of selected miRNA targets in *cyp78a5*,*7*. Based on comprehensive genetic interaction studies we propose a model in which both enzyme classes act on a common downstream process to sustain cell fate decisions in the early embryo and the shoot apical meristem.

## Author summary

Plants continuously form new organs throughout their lifetime. This reiterative organ formation enables plants to explore their habitat for essential resources such as light, water and minerals despite their inability to move. It is also the key to their remarkable regenerative power whereby they can overcome even substantial physical injury. Shoot organ formation takes place in growing points, where pluripotent stem cells steadily deliver organ-initiating daughter cells that subsequently differentiate into various tissues. For optimal fitness, it is fundamental that daughter cells lose their stem cell identity in the right place at the right time. The carboxypetidase ALTERED MERISTEM PROGRAM1 (AMP1) suppresses stem cell identity in organ-forming daughter cells, however, its specific molecular function in this process is yet unknown. Our work functionally links AMP1 with the cytochrome P450 oxidases CYP78A5/7. We show that AMP1 and CYP78A5/7 share strongly overlapping expression patterns and that their loss-of-function leads to nearly identical cell fate defects in the tissues, in which they are expressed. Based on genetic and molecular analyses we postulate that the two enzyme classes act on a common downstream process, which maintains cell fate decisions and affects miRNA-mediated inhibition of translation and protein lipidation.

## Introduction

The development of multicellular organisms requires the maintenance of pluripotent stem cells and the coordinated differentiation of stem cell descendants into organs and tissues with specific functions. Plants contain organ-forming stem cell niches called meristems which are established during embryogenesis and allow continuous and modular growth. The shoot apical meristem (SAM) is a dome-shaped structure organized into distinct zones, which harbor cells with different functions. Pluripotent stem cells are located in the central zone and their identity is specified by an underlying organizing center (OC). Stem cell descendants displaced to the meristem periphery undergo transition to a determinate cell fate during the process of organ initiation in a ring-like morphogenetic zone. Preservation of this spatial zonation is important for meristem function [1].

Classical microsurgery as well as more recent laser ablation experiments on SAMs in different species revealed that the stem cells in the central domain control the pluripotency status of their progeny in the meristem periphery [2–6]. Destruction of the central zone triggers the re-specification of a substitutive stem cell niche from PZ cells. Thus, by a yet unknown mode the stem cell niche in the SAM actively suppresses the stem cell identity of cells in the peripheral zone. This lateral inhibition of pluripotency ensures that only one active stem cell niche (SCN) is maintained and at the same time provides a safeguard system to sustain meristem function, in case of SCN damage. However, the precise molecular mechanism mediating this process is not understood to date [5, 7].

One factor, which appears to play a central role in the lateral inhibition of pluripotency is the putative carboxypeptidase ALTERED MERISTEM PROGRAM1 (AMP1). Arabidopsis seedlings mutated in *AMP1* contain enlarged SAMs, which form ectopic stem cell niches in the meristem periphery in the presence of an intact primary stem cell population [8–10]. This suggests that AMP1 is involved in the production, transport or detection of a signal, which suppresses pluripotency in the cells of the PZ. The ectopic stem cell pool formation in *amp1* shoot meristems is rather unique and genetic analyses surprisingly revealed that AMP1 acts largely in a cytokinin and WUSCHEL-independent manner [10].

Analysis of *amp1* embryo development also revealed a defect in the maintenance of suspensor cell identity. The suspensor represents a supportive structure of differentiated cells, that anchors the embryo proper in the ovule. Suspensor cells retain a latent pluripotent state and can develop into functional secondary embryos upon abortion of the initial embryo [11]. *amp1* mutant suspensors frequently start to proliferate and form an embryonic cell mass, which becomes incorporated in the primary proembryo [12].

AMP1 belongs to the M28B family of metalloproteases [9]. The best-studied member of this family is the human glutamate carboxypeptidase II (HsGCPII), which serves as a tumor marker. In neuronal tissues, GCP II de-glutamylates the neurotransmitter NAAG, whereas in the intestine the enzyme acts on poly-glutamylated folates [13]. However, its precise physiological function during normal development and tumor formation is not well understood [14]. HsGCPII and AMP1 share the same domain structure and the zinc-binding catalytic center, however the overall sequence similarity is only 28% and important residues required for GCPII substrate recognition are not conserved in AMP1 [15, 16]. Consistent with a functional divergence between the animal and plant proteins, heterologous HsGCPII expression did not rescue any of the *amp1* phenotypes [16].

It has previously been demonstrated that AMP1 contributes to the miRNA-dependent control of translation [17]. Loss of AMP1 function results in an over-accumulation of various tested miRNA target proteins. This defect was only weakly manifested in *amp1* single mutants, however additional elimination of the RNA DEPENDENT RNA POLYMERASE6 (RDR6) or the AMP1 paralog LIKE AMP1 (LAMP1) caused strongly elevated protein levels of miRNA sensitive reporters in the absence of increased mRNA levels. These data imply that AMP1/LAMP1 mediate miRNA-dependent translational inhibition without affecting miRNA-triggered mRNA slicing. Furthermore, AMP1/LAMP1-dependent translational repression was only apparent at ribosomes of the endoplasmic reticulum where AMP1 partially co-localizes with AGO1 [17]. Interestingly, the unique developmental phenotype of *amp1* is neither found in mutants with a general deficiency in the miRNA pathway [18] nor present in plant lines with a specific defect in miRNA-dependent translational repression [19–21]. This discrepancy might be caused by a locally restricted release of translational repression in the AMP1/LAMP1 expression domain affecting only a subset of miRNA targets. Recently, indirect evidence was provided that the over-accumulation of the miRNA-controlled HD-ZIP III proteins contributes to the shoot phenotype of *amp1* [22]. However, several other miRNA-targets have been shown to be mis-regulated in the mutant and their phenotypic impact has yet to be revealed.

Mutation of the *AMP1* ortholog *PLASTOCHRON3* (*PLA3*) in rice also results in a shoot hypertrophy defect with bigger SAM size, ectopic stem cell pool formation and strongly accelerated leaf formation rate [23]. The rice *pla3* mutant is highly reminiscent of *plastochron1 (pla1)*, which has a defect in the cytochrome P450 oxidase CYP78A11 [24]. CYP78A is a conserved plant specific clade of cytochrome P450 enzymes [25]. The majority of the CYP78A genes were first discovered due to their flower and meristem specific expression pattern [26–28]. In Arabidopsis, the CYP78A clade consists of 6 members. On the one hand, they have been assigned a key function in the growth control of organs, such as leaves, petals, seeds, fruits [29–32]. In this context the enzymes appear to act in a non cell autonomous manner [33]. On the other hand, loss of CYP78A5/7 function in Arabidopsis has been shown to significantly shorten the plastochron as found for the *pla1* mutant in rice [24, 34]. Since this enzyme clade oxidizes short chain fatty acids *in vitro* [35, 36], it was postulated that it might act on the production of a novel hormone like signal, which controls organ size and leaf formation rate, however the *in vivo* enzymatic functions of CYP78A clade members have yet to be discovered.

Due to the reported similarity of plastochron defects between *amp1* and *cyp78a* mutants in Arabidopsis and rice we asked whether CYP78A function also impacts cell fate maintenance and thus contributes to a common molecular pathway with AMP1. We found that AMP1 and CYP78A5 show strongly overlapping tissue-specific expression patterns in the embryo and vegetative shoot. Mutation of *cyp78a5*,*7* moreover caused the same type of suspensor-to-embryo conversion and ectopic stem cell pool formation in the shoot meristem as described for *amp1*. The phenotypic similarities were underlined by overlapping molecular signatures including expression of stem cell markers, transcriptomic changes and accumulation of AMP1-controlled miRNA targets. Finally, genetic interaction studies revealed that both enzyme classes act on a common downstream process in a partially redundant manner.

## Results

### AMP1 and CYP78A5 show overlapping expression patterns in the embryo and in the post-embryonic shoot meristem

**Fig 1:**
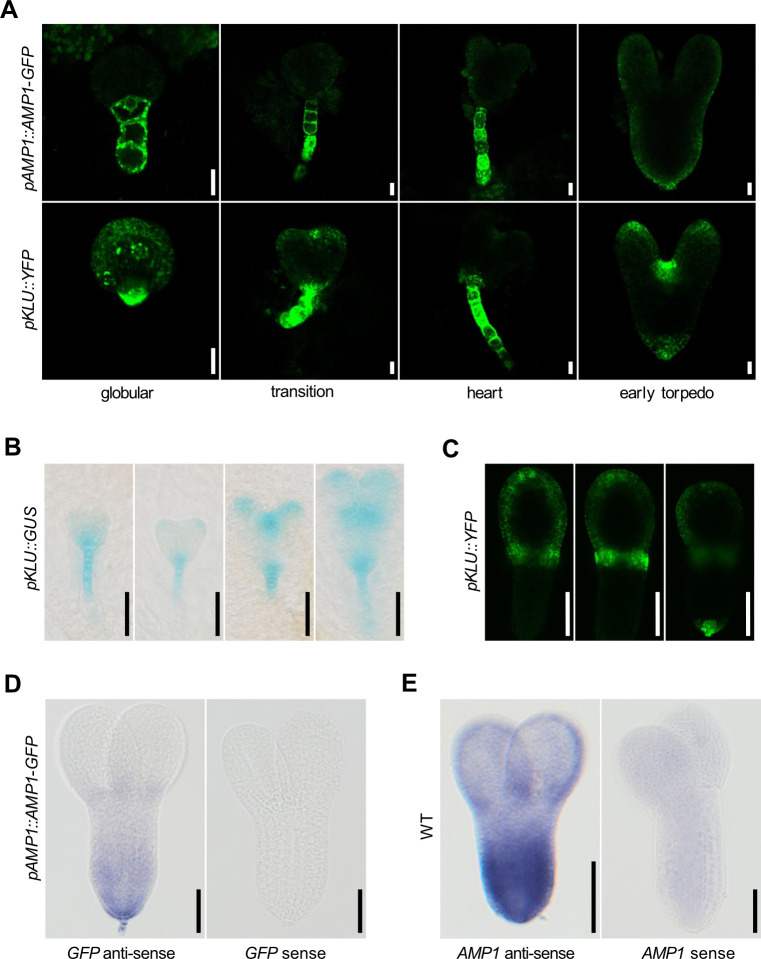
Comparison of *AMP1* and *CYP78A5* expression domains in the embryo. **(A)** pAMP1::AMP1-GFP and pKLU::YFP fluorescence in embryos at the indicated developmental stages. **(B)** Activity of the CYP78A5-specific pKLU::GUS reporter in embryos at the globular, transition, heart and torpedo stage. **(C)** pKLU::YFP fluorescence in a torpedo stage embryo. Three consecutive sagittal optical sections through a cotyledon primordium and the adjacent shoot meristem. **(D)** RNA *in situ* hybridization using a *GFP*-specific anti-sense (left) and sense (right) probe in *pAMP*::*AMP1-GFP* torpedo stage embryos. **(E)** RNA *in situ* hybridization using an *AMP1*-specific anti-sense (left) and sense (right) probe in *pAMP*::*AMP1-GFP* torpedo stage embryos. Size bars represent 10 μm (A) and 50 μm (B, C, D and F).

To define, to which extend AMP1 and CYP78A5 (also known as KLU [29]) are co-expressed in a tissue dependent manner, we compared their expression patterns during different developmental stages using available reporters and *in situ* hybridization analysis. pAMP1::AMP1-GFP could be earliest detected in the suspensor and the hypophysis of globular embryos and continued to be strongly expressed there until the late heart stage (Fig 1A). After degeneration of the suspensor, AMP1 expression was most prominent in the basal outer areas of the root pole. Moreover, the *in situ* hybridization experiments also revealed AMP1 expression at the lateral base and along the edges of the cotyledon primordia producing a horseshoe-like pattern (Fig 1D and 1E). Similar to AMP1, strong CYP78A5 expression could be detected in the suspensor and the hypophysis from the globular stage onward (Fig 1A and 1B). In addition, the CYP78A5 transcriptional reporter pKLU::YFP was active between and at the tips of emerging cotyledon poles at the heart stage. Microscopic analysis from different angles revealed a horseshoe-like expression pattern in the cotyledons of torpedo stage embryos with the strongest expression at the lateral bases encompassing the developing SAM (Fig 1C). Moreover, pKLU::YFP fluorescence sustained in the embryonic root pole after the suspensor underwent apoptosis (Fig 1A and 1C). Taken together, AMP1 and CYP78A5 show an extensive overlap of expression in the suspensor and proembryo.

**Fig 2:**
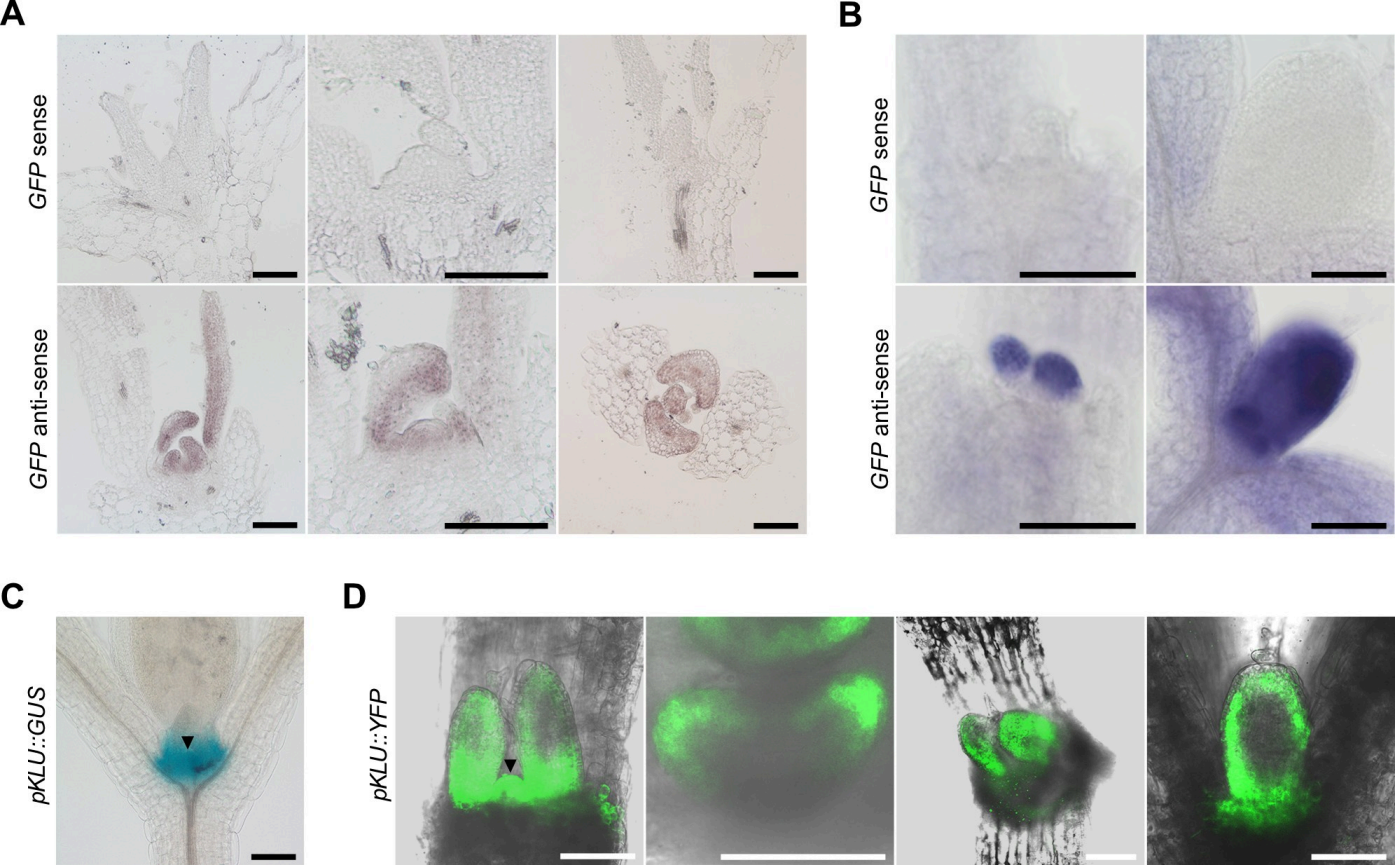
Comparison of *AMP1* and *CYP78A5* expression domains in the vegetative shoot. **(A)** RNA *in situ* hybridization with longitudinal (left, middle) and transversal (right) shoot sections of 7-day-old *pAMP1*::*AMP1-GFP* seedlings using a *GFP*-specific sense (upper panel) and anti-sense probe (lower panel). **(B)** Whole mount RNA *in situ* hybridization with shoots of 7-day-old *pAMP1*::*AMP1-GFP* seedlings using a *GFP*-specific sense (upper panel) and anti-sense probe (lower panel). In the left images the first pair of true leaves were removed. **(C)** pKLU::GUS activity in shoots of 7-day-old wild-type seedlings. The black arrowhead marks the tip of the shoot meristem area. **(D)** pKLU::YFP fluorescence in shoots of 6-day-old wild-type seedlings. Images were taken at different angles to the shoot axis; left image: coronal plane, the black arrowhead marks the tip of the shoot meristem area; two images in the middle: transversal plane; right image: sagittal plane. Size bars represent 100 μm (A, B and C) and 50 μm (D).

We also compared the expression patterns of AMP1 and CYP78A5 in the post-embryonic shoot since the corresponding mutants are particularly affected in shoot meristem function. Due to the relative weak fluorescence of the AMP1-GFP reporter in the shoot we performed RNA *in situ* hybridization analysis in this line using a GFP-specific probe. The antisense probe revealed a strong signal in young leaf primordia (Fig 2A and 2B). In longitudinal sections the signal appeared stronger at the adaxial side. Analysis of transversal sections supported a slight enhancement of the signal towards the adaxial side and the edges of the primordia (Fig 2A). Furthermore, an AMP1-GFP specific signal was also present in the shoot meristem area (Fig 2A). Analysis of pKLU::GUS and pKLU::YFP activities revealed an extensive overlap of *CYP78A5* expression with that of *AMP1* in young leaf primordia and the shoot meristem area (Fig 2C and 2D). In leaf primordia pKLU::YFP fluorescence was strongest along the rim and towards the base of the organ resulting again in a horseshoe-like pattern as found in the embryonic cotyledons. Transcriptome map mining (S1 Fig;[37]) supported substantial expression of *CYP78A5* in the border between the SAM and leaf primordia (*LAS* expression domain), in different domains of the SAM (*UFO*, *CLV3* and *WUS*) as well as in the rim region of the primordium (*PTL*). *AMP1* transcripts were also detectable in these domains but at markedly lower levels.

### Loss of CYP78A5/7 function triggers suspensor-to-embryo conversion

**Fig 3:**
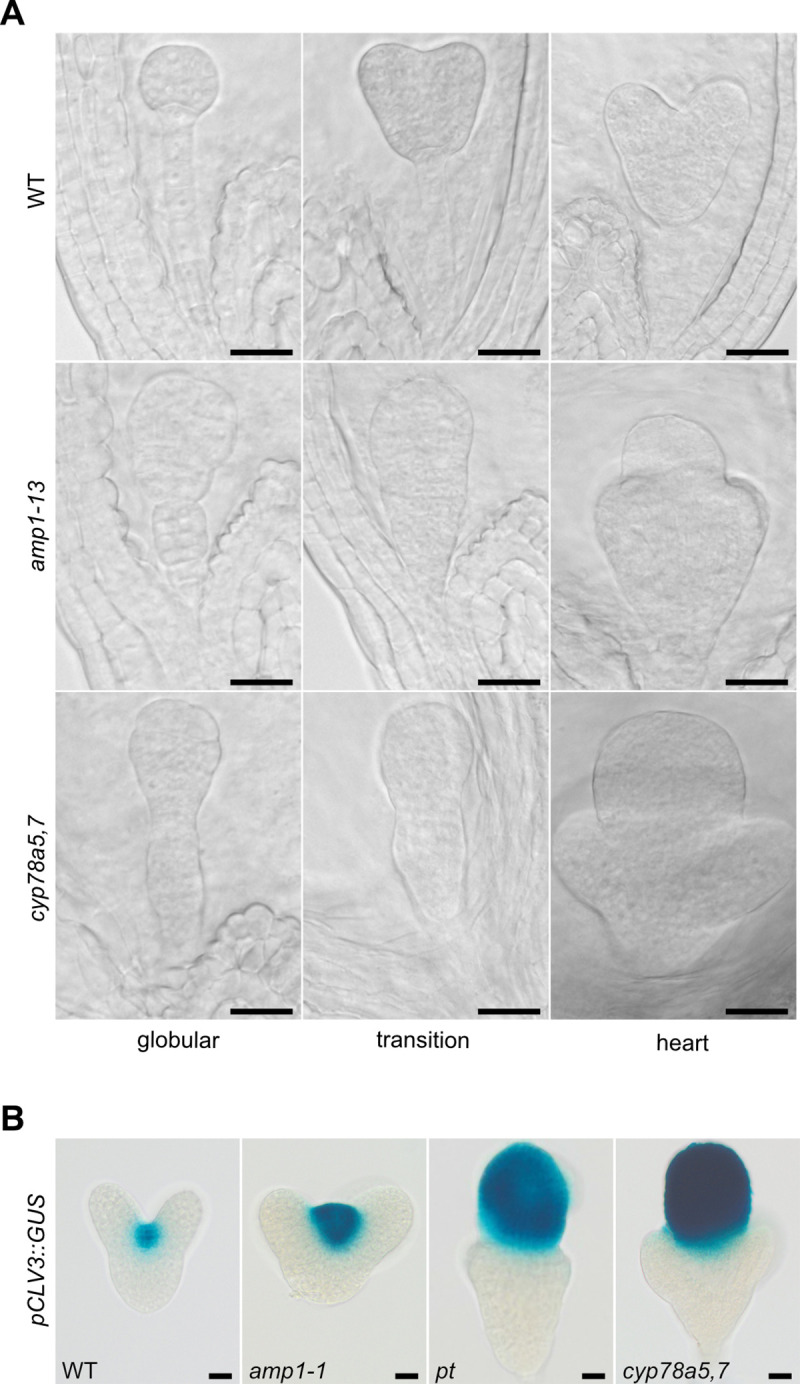
*cyp78a5*,*7* mutants show a suspensor to embryo conversion phenotype. **(A)** Wild-type (upper panel), *amp1-13* (middle panel) and *cyp78a5*,*7* (lower panel) embryos at the indicated developmental stages. **(B)** pCLV3::GUS activity in embryos of the indicated genotypes at the late heart stage. Size bars represent 20 μm.

Next we determined whether *amp1*-specific defects in cell fate control occur in CYP78A loss-of-function mutants. Since it was reported that CYP78A7 acts partially redundant with CYP78A5 in the control of the plastochron [34] and is the only homolog with a considerably overlapping expression in the SAM (S1 Fig), we generated a *cyp78a5*,*7* double mutant and analyzed its embryonic development. As previously described [12], *amp1* suspensors start to proliferate, adapt embryonic identity and fuse with the proembryo, which subsequently converts to an inordinately large shoot meristem (Fig 3A). Strikingly, *cyp78a5*,*7* double mutants show the same suspensor-specific reappearance of pluripotency, resulting in conjoined twin embryos with a massively enlarged shoot meristematic structure surrounded by more than two cotyledons. Like in *amp1*, the primary proembryo fully adapts shoot meristematic identity as indicated by the strong expression of the pCLV3::GUS reporter in this structure (Fig 3B).

### Loss of CYP78A5/7 function phenocopies the shoot hypertrophy defect of *amp1*

**Fig 4:**
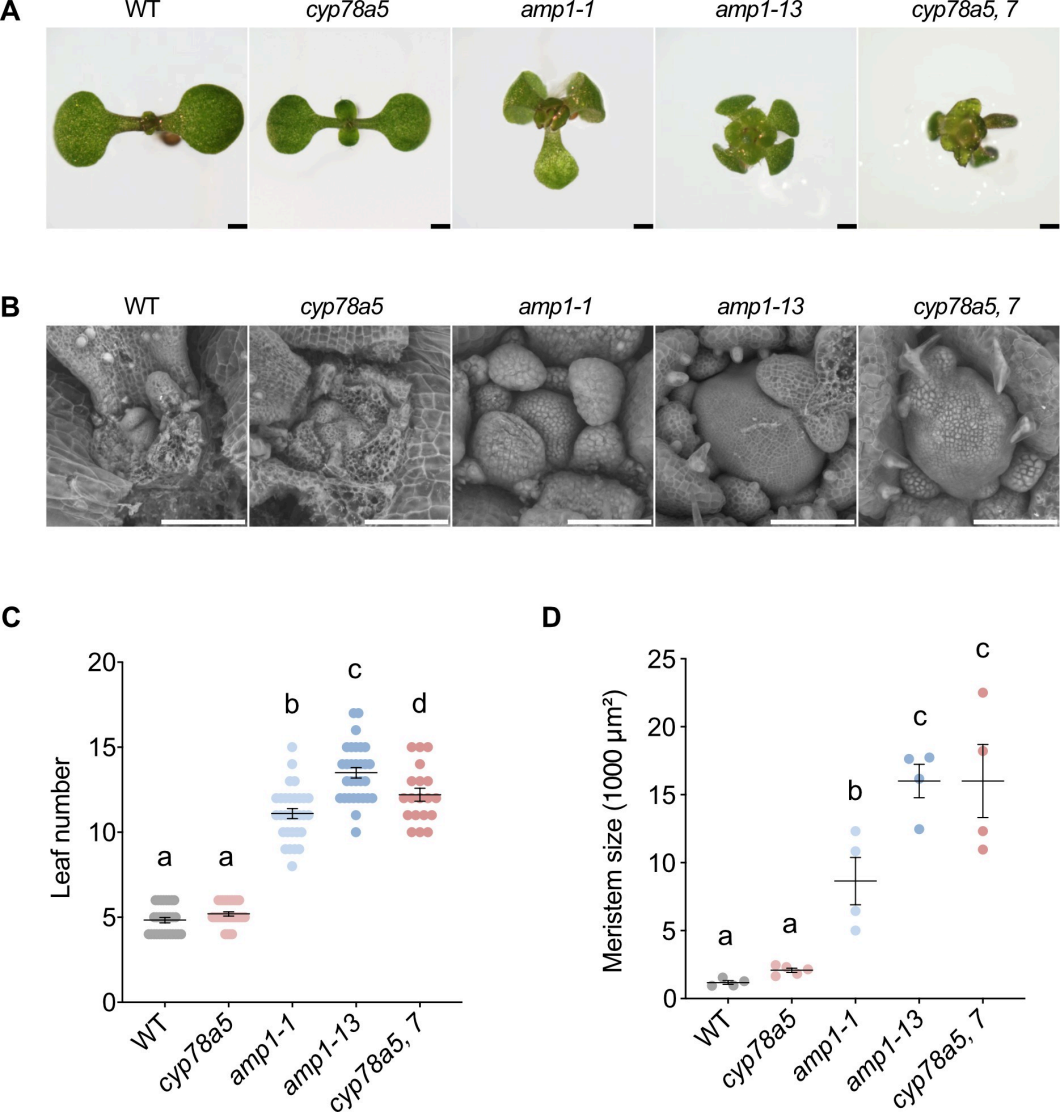
*cyp78a5*,*7* plants show *amp1*-related shoot defects. (**A**) Shoot phenotypes of wild-type, *cyp78a5*, *amp1-1*, *amp1-13* and *cyp78a5*,*7* plants at 7 DAG. (**B**) Scanning electron micrographs of shoot apices of 7-day-old wild-type, *cyp78a5*, *amp1-1*, *amp1-13* and *cyp78a5*,*7* plants. (**C**) Quantification of rosette leaf number in seedlings of the indicated genotypes at 7 DAG. (means ± SE of the mean; n ≥ 30). Different letters over the error bars indicate significant differences (P < 0.05; one-way ANOVA followed by Tukey’s multiple comparison tests). (**D**) Quantification of a SAM surface area of 7-day-old plants; (means ± SE of the mean; n ≥ 4). Different letters over the error bars indicate significant differences (P < 0.05; one-way ANOVA followed by Tukey’s multiple comparison tests). Size bars represent 500 μm (A) and 100 μm (B).

After germination, the oversized SAM of *amp1* seedlings, established during embryogenesis, further enlarges by the formation of ectopic stem cell pools in the SAM periphery [10]. The resulting stem cell cluster give rise to a massive SAM surface area and develop leaves in parallel, partially contributing to the increased leaf formation rate of the mutant (Fig 4A and 4C). *cyp78a5* seedlings only showed a slight increase in leaf number and SAM surface size compared to wild type (Fig 4A–4D). In contrast, *cyp78a5*,*7* double mutants developed a shoot morphology nearly indistinguishable to the strong *amp1-13* allele including polycotyly, precocious and pronounced formation of compact true leaves and an oversized shoot meristem area (Fig 4A and 4B). With a 2.6-fold increase in leaf number and a 12-fold increase in SAM surface compared to wild type also the quantitative severity of the *cyp78a5*,*7* defect was very close to that of *amp1-13* (Fig 4C and 4D).

### Loss of CYP78A5/7 function causes ectopic stem cell pool formation in the vegetative shoot meristem and enhances RAP2.6L expression

**Fig 5:**
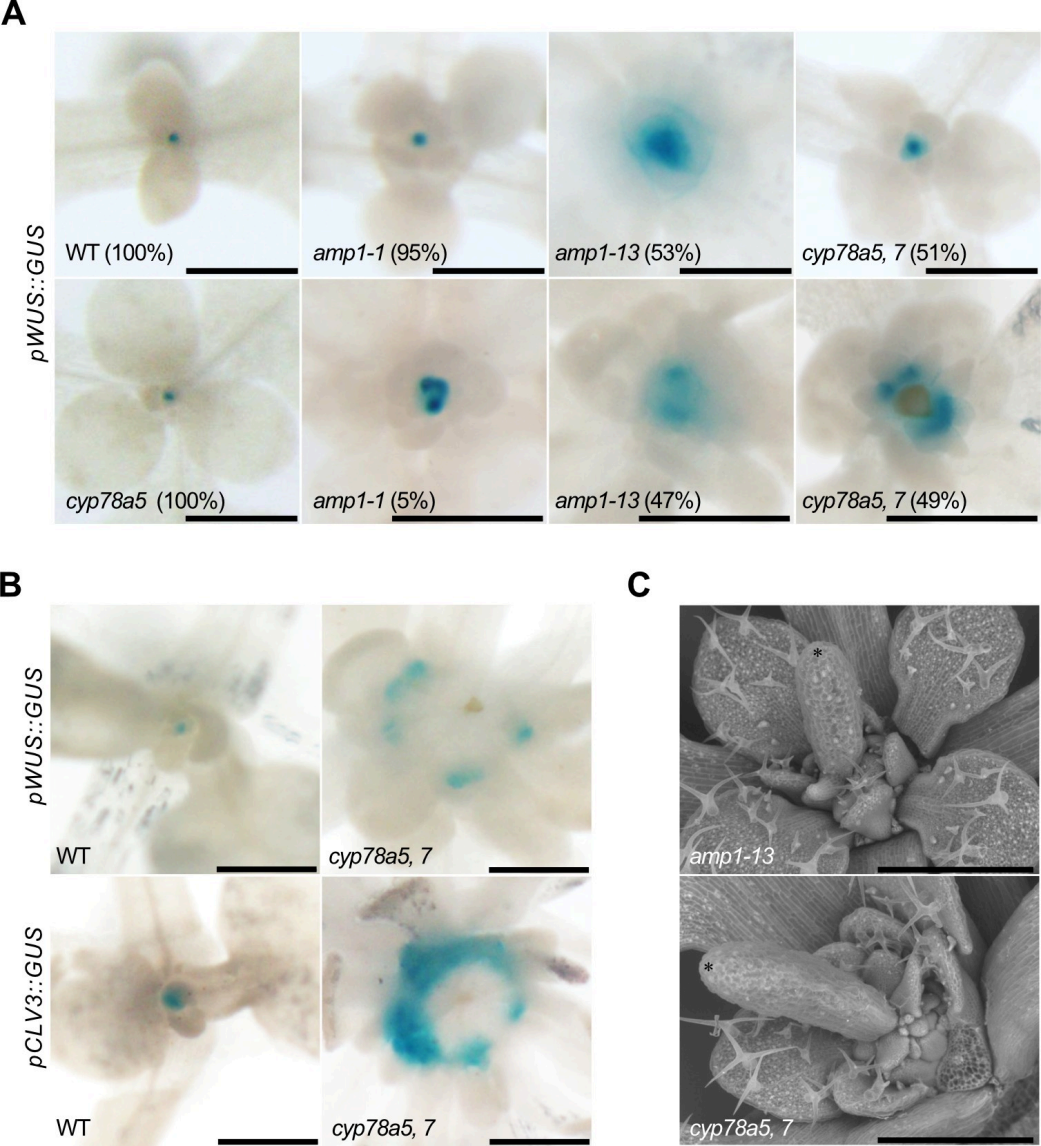
*cyp78a5*,*7* forms ectopic shoot stem cell pools similar to *amp1*. (**A**) pWUS::GUS activity in shoots of the indicated genotypes at 7 DAG. The frequencies of shown GUS-staining patterns are stated. (**B**) pWUS::GUS and pCLV3::GUS activities in shoots of wild type and *cyp78a5*,*7* at 10 DAG. (**C**) Scanning electron micrographs of shoot apices of 7-day-old *amp1-13* and *cyp78a5*,*7* plants showing the presence of a central leaf-like structure with radial polarity (black asterisk marks the tip of the structure). Leaf primordia were partially to visualize meristematic structures. Size bars represent 500 μm (A,B and C).

To follow stem cell pool development in *cyp78a5*,*7* shoot meristems, we analyzed the OC-marker pWUS::GUS in the double mutant. In *amp1*, depending on the allelic strength, between 5% (*amp1-1*) and 47% (*amp1-13*) of 7-day-old seedlings showed the formation of at least one additional WUS expression domain in the SAM periphery ([10]; Fig 5A). Ectopic OC-formation at a similar rate to that of *amp1-13* was found in *cyp78a5*,*7* seedlings, whereas *cyp78a5* single mutants were aphenotypic in this respect (Fig 5A). At a later developmental stage, the number of individual OC-foci and corresponding CLV3 expressing areas further increased in *cyp78a5*,*7* and frequently surrounded a central stem cell negative domain (Fig 5B), which often produced a radialized leaf-like organ (Fig 5C) and leaf primordia formation subsequently continued to the inner and outer side of the concentrically arranged stem cell pools. Notably, this specific SAM patterning defect is also a hallmark of strong *amp1* alleles such as *amp1-13* and *pt* ([10]; Fig 5C). These data indicate that CYP78A5/7, like AMP1, suppress the reappearance of stemness in cells of the SAM periphery.

Another specific molecular signature connected to the stem cell specification defect of *amp1* is the enhanced expression of the AP2 transcription factor RAP2.6L, which contributes to the SAM hypertrophy and enhanced regeneration potential of the mutant [22]. GUS-reporter analysis revealed a comparable prominent up-regulation of RAP2.6L expression in *cyp78a5*,*7* seedlings (S2 Fig) indicating that also this defect is conserved between the mutant lines.

### *cyp78a5*,*7* and *amp1* seedlings show significantly overlapping transcriptomic changes

**Fig 6:**
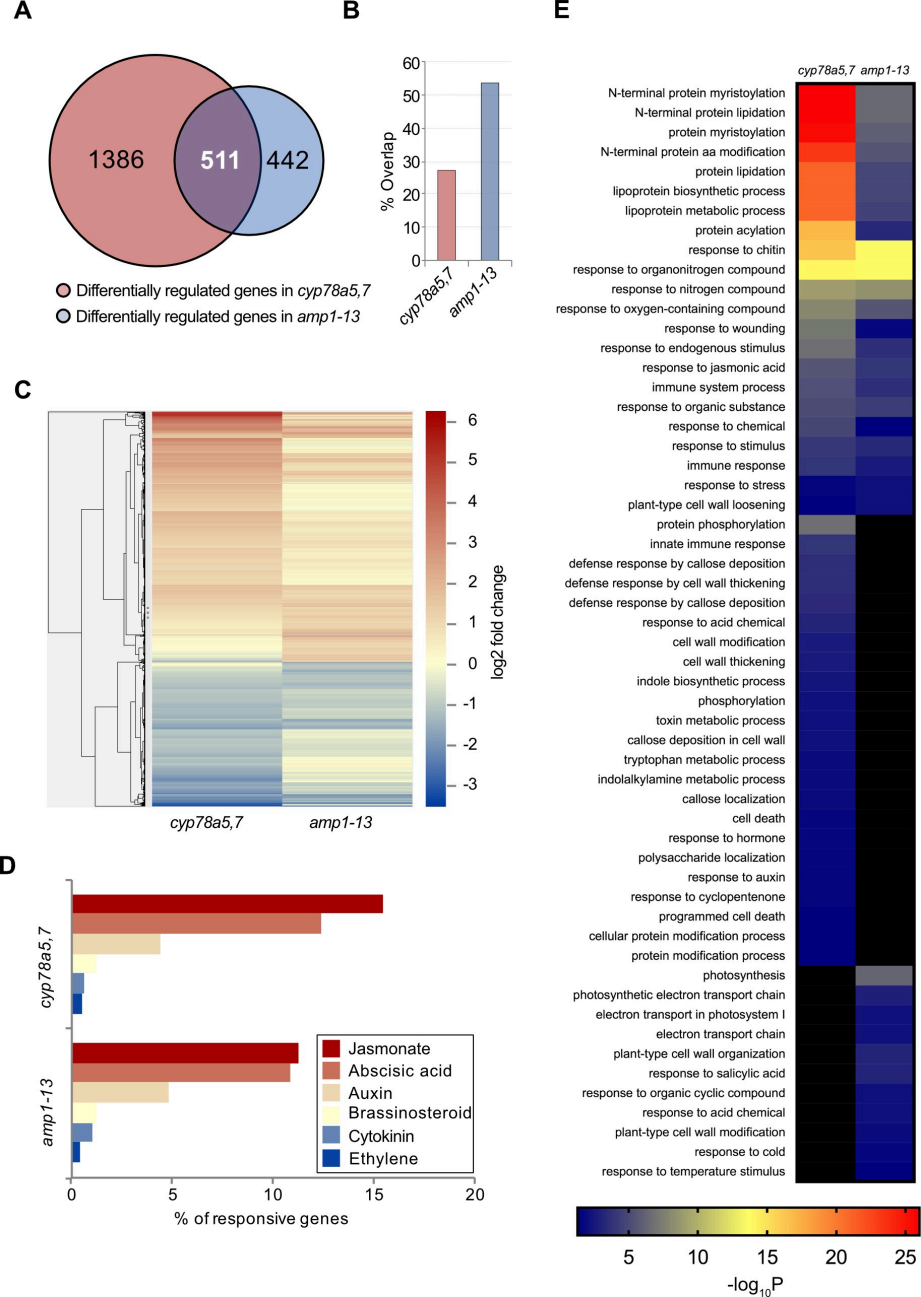
Comparative analysis of *cyp78a5*,*7 and amp1-13* transcriptomic responses. **A)** Venn diagram showing the number of genes that were differently regulated in *cyp78a5*,*7* and *amp1-13* seedlings in comparison with Col-0. **B**) Graphical representation of the overlapping portion of differentially regulated genes in *amp1-13* and *cyp78a5*,*7*. **(C)** Heat map of genes differently regulated in *cyp78a5*,*7 and amp1-13* (2339 DEGs). The heat map was produced by clustering the normalized values using the hierarchical clustering algorithm implemented in Gene Cluster (Euclidean distance and Average linkage). The results were visualized using TreeView3. **(D**) Graph showing the fractions of hormone-specific marker genes differently regulated in cyp78a5,7 and *amp1-13*. **(E)** GO term enrichment analysis of genes differently regulated in *cyp78a5*,*7 and amp1-13* (2339 DEGs).

To further assess whether these striking phenotypic similarities between *amp1* and *cyp78a5*,*7* are also depicted in a congruency at the global gene expression level, we analyzed the transcriptomic responses of both genotypes using microarray analysis. The experiment was performed with 10-day-old seedlings. Compared with wild type Col-0, 1897 and 953 genes were differentially expressed in *cyp78a5*,*7* and *amp1-13*, respectively. Of these genes, 511 were differentially expressed in both mutant lines (27% of *cyp78A5*,*7* mis-regulated genes and 54% of *amp1-13* mis-regulated genes), an overlap that is greater than would be expected by chance (P < 2.009e-308; Fig 6A and 6B; S1 Table). Moreover, expression heatmap analysis revealed that the co-regulated DEGs were largely altered in the same direction with only eight genes showing opposite change of expression between the two mutant genotypes (Fig 6C; S1 Table). To determine, whether the DEGs of the two lines show any specific hormonal signatures, we assessed the relative presence of core responsive gene sets for six different hormones (Fig 6D). Notably, for both genotypes the highest overlap was found among the set of jasmonate-responsive genes (16% present in *cyp78a5*,*7* sample, 12% present in the *amp1-13* sample). Furthermore, over 10% of ABA-responsive genes were present in the DEG sets of both mutants.

The lists of genes differently expressed in *cyp78a5*,*7* and *amp1-13* were further used in a gene ontology (GO) enrichment analysis to determine biological processes specifically affected in one or both genotypes. GO terms related to lipoprotein synthesis/metabolism, response to jasmonate/wounding and response to organic substance/chemicals were highly enriched among the 1897 *cyp78a5*,*7*-regulated genes and 953 *amp1*-regulated genes (Fig 6E). GO terms only enriched among the *cyp78a5*,*7*-regulated genes included cell wall specific processes, indole/tryptophan metabolic processes, response to auxin and programmed cell death. Finally, GO terms only enriched among the *amp1*-regulated genes were related to photosynthesis, response to salicylic acid and response to temperature stimulus. Taken together, defects in CYP78A5/7 and AMP1 cause a substantially overlapping read out at the global gene expression level, supporting a model in which both enzyme types affect a common molecular process.

### AMP1/LAMP1 and CYP78A5/A7 act synergistically in shoot meristem patterning

**Fig 7:**
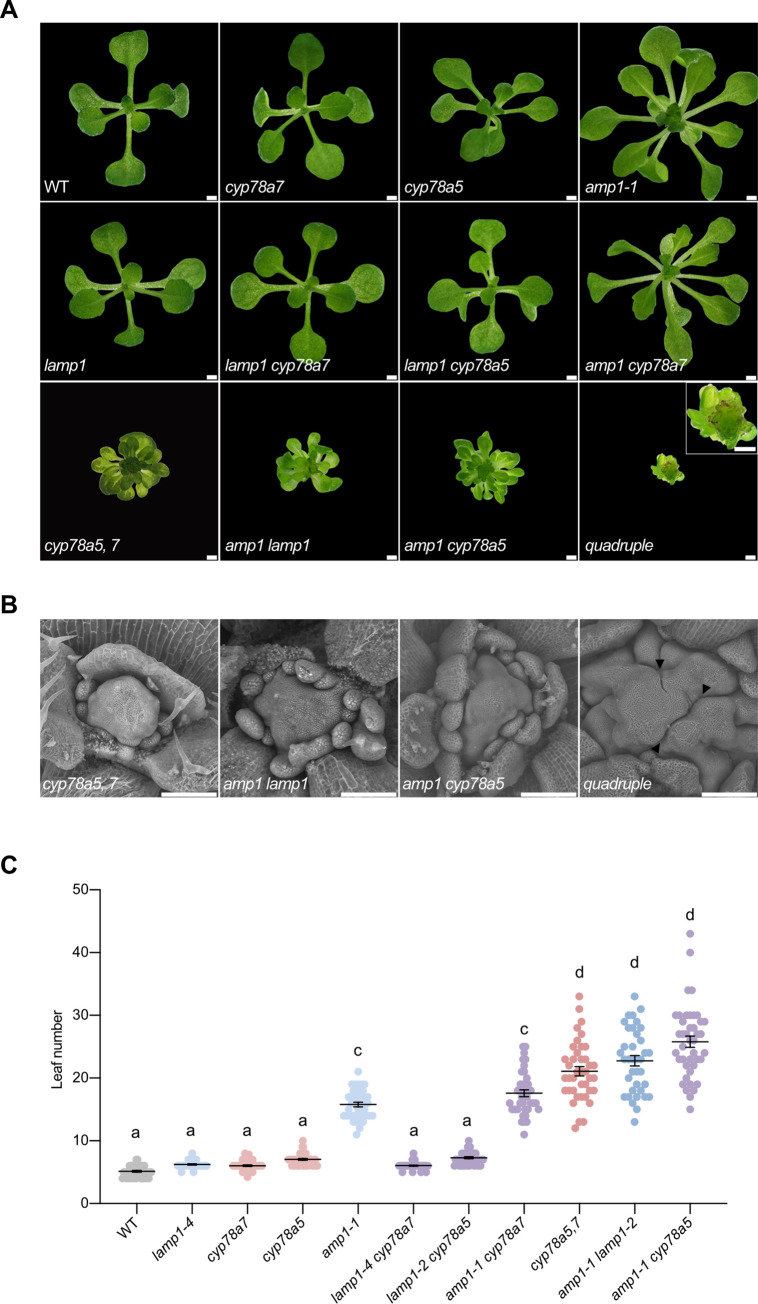
Seedling phenotypes of *amp1*, *lamp1*, *cyp78a5 and cyp78a7* mutant combinations. **(A)** Shoot phenotypes of indicated genotypes at 14 DAG (except for the quadruple mutant, which was pictured at 12 DAG). A close-up of the quadruple mutant is presented in the upper right corner. **(B)** Scanning electron micrographs from shoot apices of the indicated genotypes at 7 DAG. In *cyp78a5*,*7* and *amp1 lamp1* some of the leaf primordia were removed for better visibility of the meristem structure. Arrowheads mark the callus-like invaginations in the quadruple mutant. **(C)** Quantification of rosette leaf number in the indicated genotypes at 10 DAG (means ± SE of the mean; n ≥ 35). Different letters over the error bars indicate significant differences (P < 0.05; one-way ANOVA followed by Tukey’s multiple comparison tests). Size bars represent 1 mm (A) and 250 μm (B).

To characterize the genetic interactions between the members of the two enzyme classes we created the according double mutants and the *amp1 lamp1 cyp78a5*,*7* quadruple mutant. We then compared their phenotypes to those of the corresponding single and enzyme class-specific double mutants. To this purpose we quantified leaf number and assessed the morphological appearance of the shoot at the vegetative growth stage (Fig 7). From the double mutants, only *lamp1 cyp78a7* was wild-type like, as its single mutant parents (Fig 7A and 7C). *lamp1 cyp78a5* showed the same subtle increase in leaf number as *cyp78a5*. In a similar manner, *amp1 cyp78a7* was indistinguishable from *amp1* in terms of increased leaf number and overall appearance. Notably, combination of *amp1* with *cyp78a5* resulted in a strong synergistic phenotype resembling *amp1 lamp1* and *cyp78a5*,*7* in respect to size, shape and spatial distribution of leaves. Finally, bringing together all four mutations in *amp1 lamp1 cyp78a5*,*7* resulted in extremely dwarfed plants with a massively expanded central meristematic area surrounded by small leaf primordia, which stayed tiny and did not form leaf blades, representing a further exaggeration of the severe double mutant phenotypes (Fig 7A). Thus, accurate quantification of leaf number was not possible in the quadruple mutant. Moreover, these plants never bolted and were not viable over an extended period of time.

We also monitored the anatomical changes in SAM structure in *amp1 cyp78a5* and the enzyme class-specific double mutants and compared them to those of the quadruple mutant. *amp1 cyp78a5* shoot apices were nearly indistinguishable to those of *amp1 lamp1* showing a massive increase in size already seven days after germination with subsequent splitting in a field of meristematic clusters, which formed organ primordia in a stochastic manner (Fig 7B). *cyp78a5*,*7* shoot meristems appeared to be smaller before fragmentation compared to those of *amp1 lamp1* and *amp1 cyp78a5*. In contrast, the quadruple mutant showed a novel phenotype, where the primary SAM was even further increased in size and generated callus-like invaginations without forming clearly distinct subunits surrounded by organ anlagen as found in the analyzed double mutants (Fig 7B).

**Fig 8:**
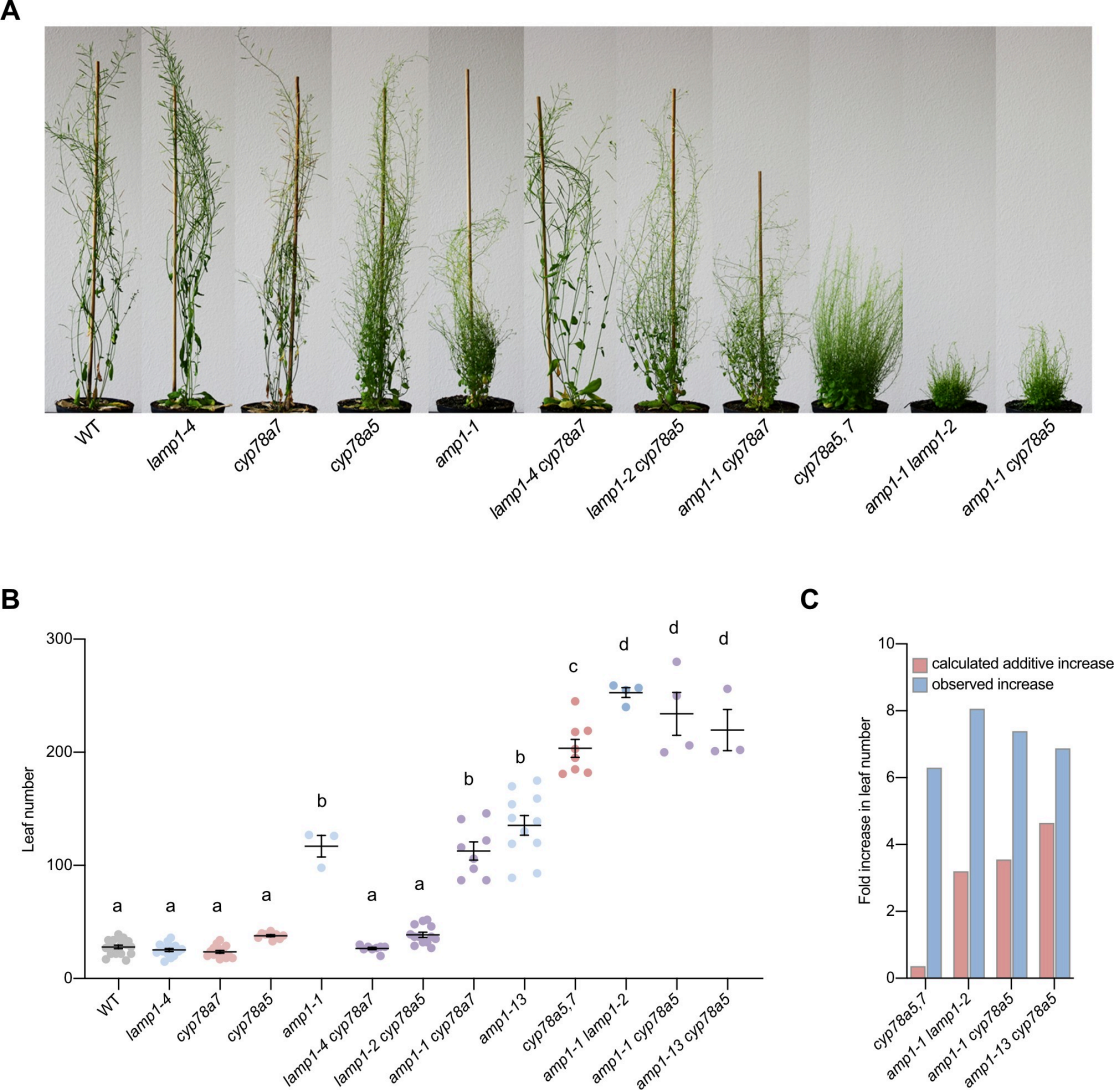
Adult shoot phenotypes of *amp1*, *lamp1*, *cyp78a5 and cyp78a7* mutant combinations. (**A**) Shoot phenotypes of indicated genotypes at 63 DAG. (**B**) Quantification of rosette leaf number at 63 DAG (means ± SE of the mean; n ≥ 4). Different letters over the error bars indicate significant differences (P < 0.05; one-way ANOVA followed by Tukey’s multiple comparison tests). (**C**) Comparison of calculated additive and observed fold increase in leaf number in the indicated double mutants compared to wild type based on the data shown in (B). Calculated additive increase: putative fold change, if the leaf number increases of the parental mutants are added up. Observed increase: the fold increase in leaf number in a double mutant in comparison to wild type.

We expanded the phenotypic analysis of mutant combinations to the adult growth stage. Like in the seedling stage, *lamp1*, *cyp78a7* and the resulting double mutant were wild-type like, whereas *cyp78a5* and *lamp1 cyp78a5* showed increased shoot branching combined with a moderately higher leaf number (Fig 8A and 8B). Shoot organ number was comparably further enhanced in *amp1-1* and *amp1-1 cyp78a7*. Plants, which lost both *AMP1* and *CYP78A5*, were super-bushy dwarfs with a massively increased leaf number and ranked phenotypically between *cyp78a5*,*7* and *amp1-1 lamp1-2*. All three double mutants were also fully sterile. The observed increase in leaf number in the double mutants clearly exaggerated the sum of supernumerary leaves in the corresponding parental single mutants (Fig 8C). To determine the impact of residual AMP1 function in *amp1-1* on this analysis we also combined the putative null allele *amp1-13* with *cyp78a5*. This line did not show a further enhancement of the adult shoot phenotype compared to *amp1-1 cyp78a5* (Fig 8B and 8C). Taken together, we observed a clear synergistic increase in phenotypic severity when combining *amp1* and *cyp78a5*, which was further pronounced in the *amp1 lamp1 cyp78a5*,*7* quadruple mutant.

### Ectopic expression of *LAMP1* rescues *amp1*

Based on the presented and earlier genetic interaction studies [10, 34], the two analyzed CYP78 members as well as both GCPII-like proteins appear to act in a partially redundant manner. In both cases there is one master paralog (CYP78A5 and AMP1), which shows a single mutant phenotype and a supporting paralog (CYP78A7 and LAMP1) whose function becomes visible only, if the master paralog is missing. Such a hierarchy would for example occur, if both paralogs are functionally conserved but the master paralog shows a broader spatiotemporal expression pattern than the supporting paralog, as indicated by our previous tissue-specific expression analysis of AMP1 and LAMP1 [10]. To test this hypothesis, we expressed a YFP-tagged version of LAMP1 under the control of the 35S promoter in *amp1-1*. Several independent *35S*::*LAMP1*:*YFP* lines were generated and characterized in the wild-type and *amp1* mutant background. In the wild-type background, *35S*::*LAMP1*:*YFP* did not show any obvious growth phenotype except of a slightly increased leaf number at 7 DAG (S3 Fig). When brought into *amp1-1*, *35S*::*LAMP1*:*YFP* progressively rescued the mutant-associated phenotype (S3 Fig). Whereas at 7 and 9 DAG both analyzed lines showed an intermediate leaf number, significantly lower than *amp1-1* but still around four leaves more than the wild-type control, this difference fully disappeared at 15 DAG. From this time point on *35S*::*LAMP1*:*YFP amp1-1* behaved like wild type, showing a normal flowering time and shoot architecture. The gradual nature of this phenotypic rescue might reflect the activity of the selected promoter, since it is known that 35S driven expression is only weak during embryogenesis when AMP1-deficient plants already establish a bigger SAM and true leaf primordia (Fig 3; [38]). Taken this into account, we conclude that LAMP1 can take over AMP1 function when its expression domain is expanded.

### CYP78A5 overexpression diminishes SAM size and leaf formation rate

To analyze the effect of *CYP78A5* overexpression (OE) on vegetative SAM function, we generated myc-tagged and untagged versions of *CYP78A5* under the control of the 35S promoter. Overexpression of the transgenic protein in *35S*::*CYP78A5*:*MYC* was verified by western blotting (S4E Fig). To determine vegetative shoot meristem activity in these lines we assessed leaf number and SAM anatomy. Both *CYP78A5* OE lines showed a retarded outgrowth of the first pair of true leaves and continued to form less leaves compared to wild type at later vegetative growth stages (S4A and S4C Fig). The reduced leaf initiation rate of *CYP78A5* OE lines correlated with a significant decrease of SAM size (S4B and S4D Fig). The SAM surface area reached only 35% (*35S*::*CYP78A5*) and 56% (*35S*::*CYP78A5*:*MYC*) of that of wild type, respectively. Taken together, enhanced CYP78A5 activity results in a decrease of leaf initiation rate and vegetative SAM size indicating a rate limiting function in the control of these parameters.

### *CYP78A5* overexpression suppresses *amp1* mutant shoot phenotypes

**Fig 9:**
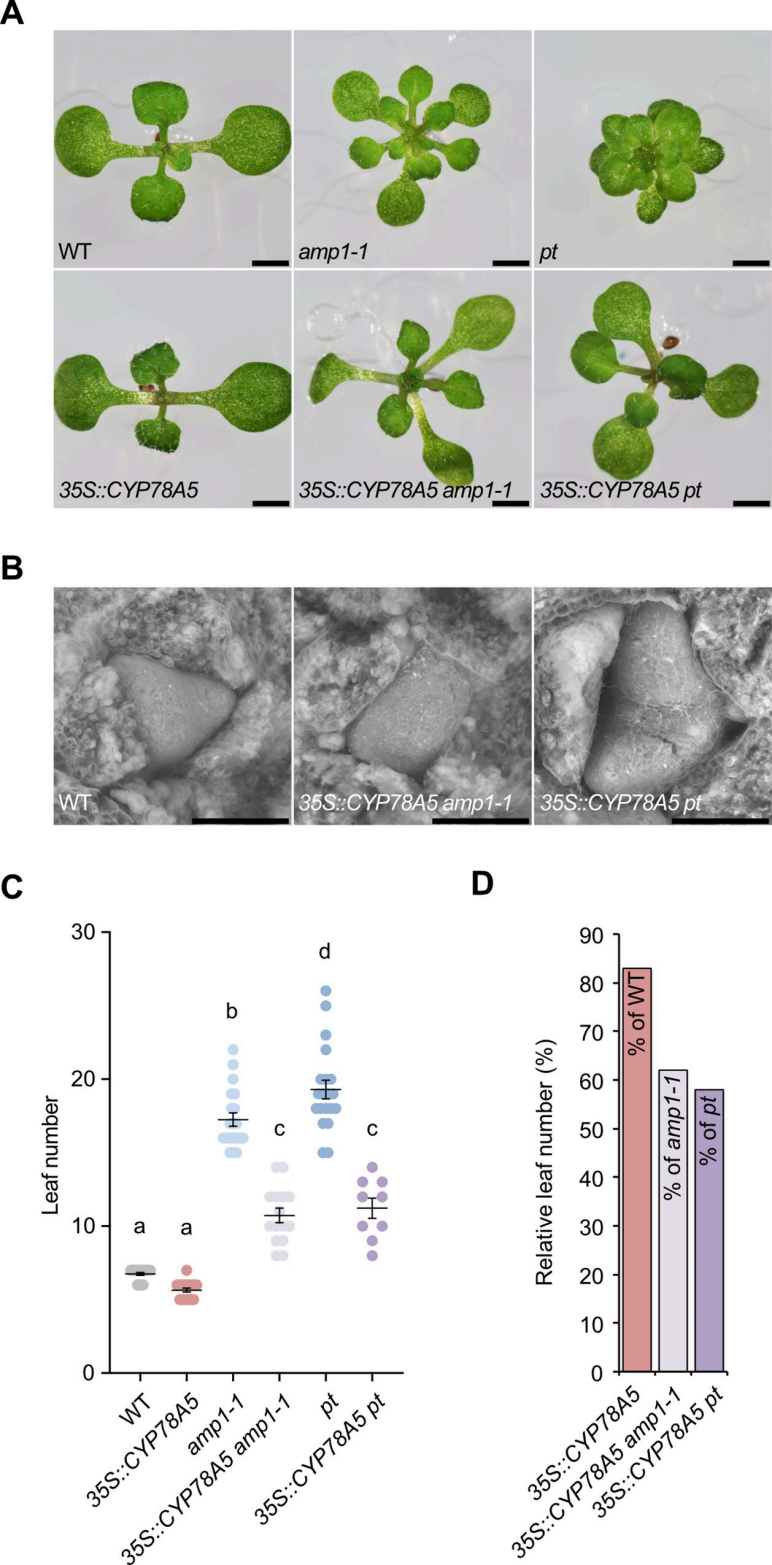
*CYP78A5* overexpression suppresses *amp1* shoot phenotypes. (**A**) Seedling shoot phenotypes of indicated genotypes at 10 DAG. (**B**) Scanning electron micrographs of shoot meristems from 10-day-old plants of indicated genotypes. **(C)** Quantification of rosette leaf number in the indicated genotypes at 10 DAG. (means ± SE of the mean; n ≥ 10). Different letters over the error bars indicate significant differences (P < 0.05; one-way ANOVA followed by Tukey’s multiple comparison tests). **(D)** Graph showing leaf numbers of CYP78A5 over-expressing lines normalized against the respective non-transgenic parental genotypes. Size bars represent 1 mm (A) and 50 μm (B).

The results of the genetic interaction analysis between the AMP1 class of proteases and the two CYP78A members are consistent with a model in which both protein classes act on a common downstream process controlling shoot meristem organization. To further test this model we determined the effect of *35S*::*CYP78A5* overexpression on the shoot phenotypes of *amp1-1* and *pt*. We included *pt*, as an *AMP1* null-allele since we observed in *amp1-13* a tendency for silencing of 35S promoter containing transgenes, which might falsify the outcome of the intended experiment. Whereas *35S*::*CYP78A5* bearing *amp1-1* and *pt* seedlings still showed supernumerary cotyledons, true leaf formation was significantly decelerated and also leaf expansion and shape appeared normalized (Fig 9A and 9C). While the presence of *35S*::*CYP78A5* reduced the leaf number by 17% in wild type, it diminished the leaf number by around 40% in the *amp1* mutants (Fig 9C and 9D). Moreover, the sizes of CYP78A5 overexpressing *amp1-1* and *pt* shoot meristems closely resembled those of wild-type plants (Fig 9B). Thus, ectopic CYP78A5 expression significantly suppresses the enhanced leaf formation and SAM hypertrophy of *amp1*.

### *AMP1* overexpression does not alter shoot meristem activity in wild type and *cyp78a* mutants

**Fig 10:**
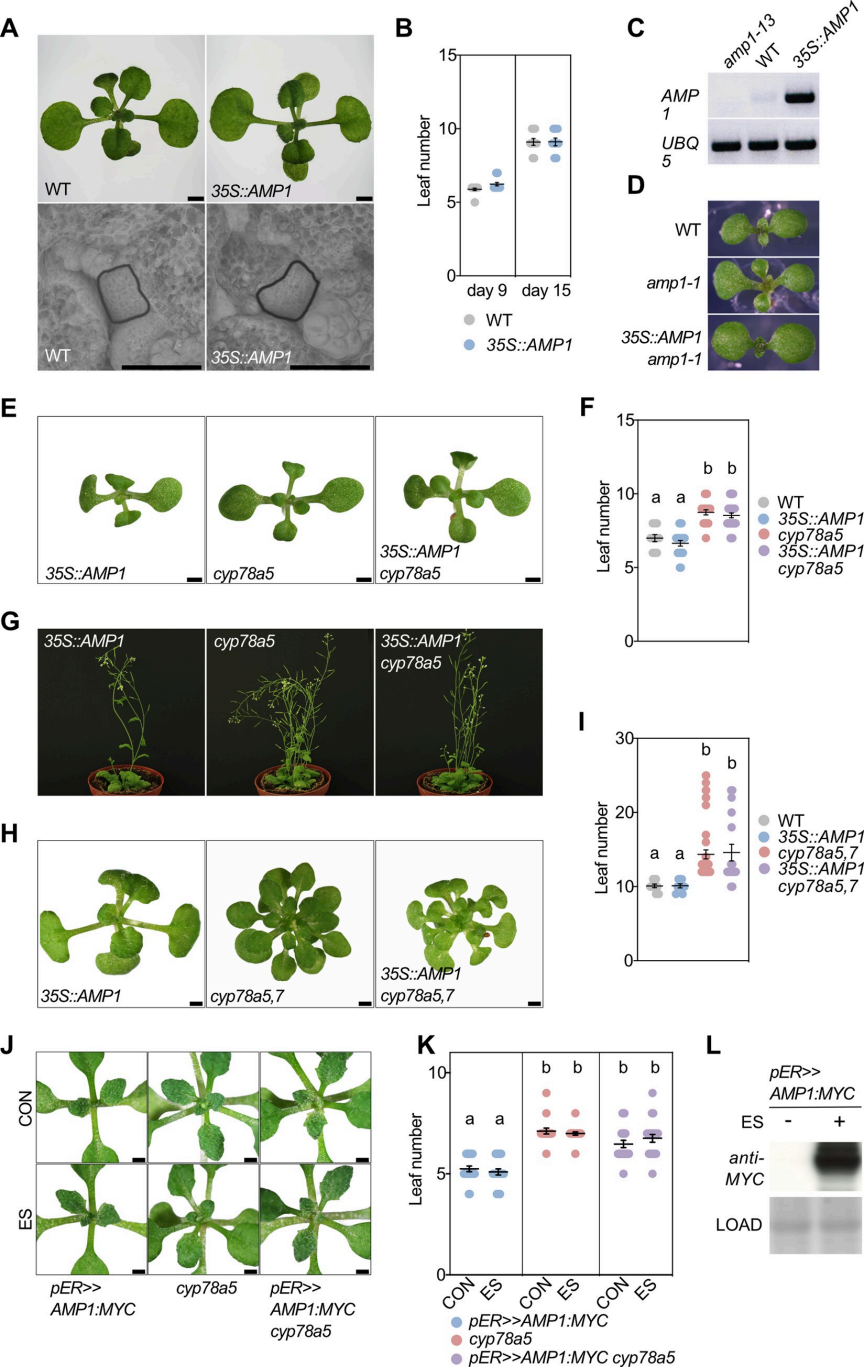
*AMP1* overexpression does not rescue *cyp78a5*,*7*-related shoot phenotypes. **(A**) Seedling shoot phenotypes of wild type and *35S*::*AMP1* at 15 DAG (upper panel) and scanning electron micrographs of their shoot apical meristems at 7 DAG (lower panel). **(B)** Quantification of rosette leaf number in wild type and *35S*::*AMP1* at the indicated time points (means ± SE of the mean; n ≥ 10). **(C)** *AMP1* expression analysis in the indicated genotypes by sqRT-PCR. Normalization of cDNA was performed with *UBQ5*-specific primers. **(D)** Seedling leaf phenotype of the indicated genotypes at 8 DAG. **(E)** Seedling shoot phenotypes of indicated genotypes at 10 DAG. **(F)** Quantification of rosette leaf number in the indicated genotypes at 10 DAG. (means ± SE of the mean; n ≥ 10). Different letters over the error bars indicate significant differences (P < 0.05; one-way ANOVA followed by Tukey’s multiple comparison tests). **(G)** Adult shoot phenotypes of indicated genotypes at 35 DAG. **(H)** Seedling shoot phenotypes of indicated genotypes at 12 DAG. **(I)** Quantification of rosette leaf number in the indicated genotypes at 17 DAG. (means ± SE of the mean; n ≥ 10). Different letters over the error bars indicate significant differences (P < 0.05; one-way ANOVA followed by Tukey’s multiple comparison tests). **(J)** Seedling shoot phenotypes of indicated genotypes at 15 DAG in the absence (CON) and presence of 10 μM estradiol (ES). **(K)** Quantification of rosette leaf number in the indicated genotypes at 9 DAG germinated either in the absence (CON) or presence (ES) of 10 μM estradiol (means ± SE of the mean; n ≥ 10). Different letters over the error bars indicate significant differences (P < 0.05; one-way ANOVA followed by Tukey’s multiple comparison tests). **(L)** Immunoblotting of protein extracts of 10-d-old *pER>>AMP1*:*MYC* seedlings grown in the absence or presence of 10 μM estradiol. AMP1:MYC was detected using an anti-MYC antibody. Ponceau S staining of the blot is shown as loading control. Size bars represent 2 mm (A) 1 mm (B and G).

To further investigate the nature of the AMP1/CYP78A functional interaction, we monitored the shoot development of a transgenic line bearing the AMP1 genomic DNA fused to the 35S promoter (*35S*::*AMP1*). This line showed increased *AMP1* transcript levels (Fig 10C) and fully rescued the *amp1-1* mutant phenotype (Fig 10D). *35S*::*AMP1* plants had the same leaf number and the same SAM size as wild type and did not exert any other obvious shoot phenotypes under the used standard growth conditions (Fig 10A and 10B). Next, we crossed *35S*::*AMP1* with *cyp78a5* and *cyp78a5*,*7*. Double homozygous *35S*::*AMP1 cyp78A5* plants were indistinguishable from *cyp78a5* parents at the seedling as well as the adult developmental stage (Fig 10E–10G). Similarly, *35S*::*AMP1* did not obviously alter the leaf over-accumulation phenotype of *cyp78a5*,*7* mutants (Fig 10H and 10I). We also crossed the estradiol-inducible AMP1 overexpression line *pER>>AMP1*:*MYC* with *cyp78a5* and analysed the leaf formation rate of seedlings germinated in the presence and absence of estradiol (Fig 10J–10L). Induction of AMP1 overexpression did not significantly suppress the higher leaf number of 9-d-old *cyp78a5* seedlings.

### *cyp78a5*,*7* shows elevated protein levels of the miRNA targets PHV and AGO1

AMP1/LAMP1 deficiency results in protein over-accumulation of various tested miRNA targets, without affecting their transcription levels [17]. To test whether *cyp78a5*,*7* also shares this specific molecular defect with *amp/lamp1*, we assessed the protein levels of the miRNA165/166 targeted HD-ZIP III transcription factor PHV using an YFP-tagged version driven by the 35S promoter, which has been shown to over-accumulate in *amp1* [16]. Using three independent biological repeats we found significant higher PHV-GFP protein levels in *cyp78a5*,*7* compared to wild type (S5A and S5C Fig). To also monitor the protein levels of a miRNA target transcribed at endogenous levels, we further analyzed the expression of the slicer protein AGO1, which is under control of miRNA168. Using an AGO1-specific antibody, we detected approximately two-fold more AGO1 protein in *cyp78a5*,*7* compared to wild type (S5B and S5D Fig), which is an increase in a similar range as reported for *amp1/lamp1* [17]. These data imply that not only AMP1/LAMP1 but also CYP78A5/7 affect protein levels of miRNA targets.

## Discussion

The putative carboxypeptidase AMP1 plays a central role in cell fate maintenance in different developmental contexts, however its molecular function in this process remains enigmatic. To broaden the AMP1 regulatory network we set out to characterize additional factors potentially acting in the same or overlapping pathways with the protease. Here we show that CYP78A5 is expressed in the same embryo and shoot apex domains as AMP1. Loss of CYP78A5 and its homolog CYP78A7 provokes a set of cell fate defects in the embryo and shoot meristem highly reminiscent of those found in *amp1*. These phenotypic similarities are accompanied by an overlap of molecular alterations, including mis-expression of stem cell markers, global transcriptional changes and protein over-accumulation of miRNA target proteins. Moreover, we observed a strong synergistic phenotypic enhancement in inter-family mutant combinations. Based on these findings we propose that the analyzed members of the two enzyme families act in or on a common pathway of cell specification controlling pluripotency in the embryo and shoot meristem.

Phenotypic comparison of single, double and quadruple mutants revealed a partially redundant functional interaction inside as well as between enzyme family members. For both enzyme classes there is a master paralog (AMP1 and CYP78A5) showing a single mutant phenotype and a supporting paralog (LAMP1 and CYP78A7), whose function only becomes apparent if the master paralog is missing. Currently we favor a model in which this hierarchy mainly results from different spatiotemporal expression patterns of master and supporting paralogs, whose biochemical functions are conserved. This model is consistent with our tissue-specific expression analysis of *AMP1* and *LAMP1*. *LAMP1* is mainly expressed in vascular-associated tissues [10], whereas *AMP1* expression is detected in vascular strands, leaf primordia, meristematic and embryonic tissues (Figs 1 and 2; [10, 12]). Moreover, ectopic expression of *LAMP1* suppressed *amp1*-associated shoot phenotypes further supporting that AMP1 and LAMP1 have equivalent biochemical activities and that the partial functional redundancy is caused by their different expression domains. In the case of *CYP78A5/A7*, both genes are expressed in the shoot meristem with *CYP78A5* at a significant higher level than *CYP78A7* (S1 Fig). Enzymatic profiling further revealed similar substrate specificities of recombinant CYP78A5 and CYP78A7 when tested against various fatty acids [36], however, functional comparison *in vivo*, for example by promoter swap experiments, has yet to be performed.

Our analysis further revealed clear synergistic genetic interactions between enzyme family members cumulating in the severe phenotype of the *amp1 lamp1 cyp78a5*,*7* quadruple mutant. In our opinion the observed genetic relationships fit best to a scenario, in which AMP1/LAMP1 and CYP78A5/7 act in parallel pathways, which converge on a common downstream node rather than acting consecutively in a linear pathway. If one enzyme class is compromised the other, to a certain extent, is still able to independently maintain the required downstream function. This model is also consistent with the strong up-regulation of CYP78A5 expression in *amp1* (S1 Table; [9, 16]) and the partial rescue of the *amp1* shoot phenotype by *CYP78A5* overexpression (Fig 9). In contrast, AMP1 overexpression did not result in a comparable phenotype to that of CYP78A5 overexpression and did not ameliorate the *cyp78a5*,*7* phenotype (S3 Fig; Fig 10). We did also not find significant up-regulation of *AMP1* expression in the *cyp78a5*,*7* double mutant (S1 Table). Thus, AMP1 seems to be required for proper function of the pathway, but does not play a rate-limiting role in this context. Notably, our results fit to the double mutant analysis of rice orthologs *pla1* (CYP78A11) and *pla3* (OsAMP1), which also revealed a synergistic rather than epistatic or additive relationship in the control of the plastochron [23].

What is the identity of the downstream process corporately regulated by AMP1/LAMP1 and CYP78A5/7? It is tempting to speculate that CYP78A5/7 are involved in miRNA-mediated repression of translation in which AMP1 has been shown to play a central role (Li et al., 2013). In an initial analysis we indeed found in *cyp78a5*,*7* enhanced protein levels for two selected miRNA targets, reported to be up-regulated, if AMP1/LAMP1 function is lost [16, 17], which supports a general role in this process. However, in regard to plastochron control, *cyp78A5*,*7* rather resembles plants with increased miRNA156/157 activity or diminished accumulation of their targets such as SPL9 and SPL13 [34]. Moreover, expression of a miRNA-resistant version of SPL9 accordingly suppressed the plastochron phenotype of *cyp78a5*, whereas overexpression of miRNA156f further enhanced it in an additive manner [34]. These data indicate that the plastochron phenotype of *cyp78a5*,*7* can be hardly attributed to decreased miRNA156/157 function. This is even more surprising since it was reported that these miRNAs limit SPL expression mainly at the level of translation [39].

It has to be noted that also in the case of *amp1/lamp1* it is currently unclear to which extent the observed cell fate defects are a direct result of the reported translational de-repression of miRNA targets. Suspensor proliferation has been reported for some miRNA biogenesis mutants, however this defect was usually associated with severe malformation or abortion of the proembryo [40], in contrast to the early and seamless fusion of suspensor and proembryo derived cell masses to a viable embryonic structure found in *amp1*. One possible explanation for this discrepancy might be that release of translational repression of miRNA targets is restricted to the AMP1/LAMP1 expression domain and/or affects only a subset of miRNA targets with a role in embryo and SAM patterning. The latter is supported by a recent study, in which no translational de-repression of miR156 target SPL9 or the miR159 target MYB33 could be observed in *amp1* [41]. We showed that AMP1 acts through the AP2 transcription factor RAP2.6L, which is controlled by the miRNA-regulated HD-ZIP III proteins [22] and we are currently analyzing the functional relationship between HD-ZIP III mis-regulation and *amp1* phenotypes. Notably, RAP2.6L expression is up-regulated in *cyp78a5*,*7* and coincides with enhanced protein levels of the HD-ZIP III member PHV. Thus, it cannot be excluded that AMP1 and CYP78A5 cooperate in their overlapping expression domains to regulate a subset of miRNA targets with a function in cell fate maintenance.

Alternatively, it can be speculated that AMP1/CYP78A deficiency affects miRNA-dependent translation but the defect in this process is not immediately causative for the developmental phenotypes but rather a consequence of a different molecular upstream activity also required for the cell fate control in differentiation processes. Interestingly, the microarray analysis revealed a significant enrichment of GO terms connected to protein lipidation processes such as N-myristoylation in both mutant classes. Recombinant CYP78A5 and 7 have been shown to omega-oxidize short fatty acids such as lauric acid and myrisitc acid *in vitro*, however the *in vivo* catalytic activity as well as the endogenous substrates are not known [36]. CYP78A function might affect lipid composition or membrane association of proteins and in this way might impact on miRNA-dependent translation, which has been reported to be mainly localized to the rough ER [17]. We note in this regard that Arabidopsis AGO1 has been shown to be a peripheral membrane protein and defects in isoprenoid or sterol biosynthesis compromise miRNA function and membrane association of AGO1 [42]. AMP1 partially co-localizes with AGO1 in the rough ER [17], nevertheless, whether its biochemical function is a prerequisite for proper membrane interaction of AGO1 and/or other proteins is unknown to date. Alternatively, CYP78A5 might generate a fatty acid-derived signal that controls the expression or activity of miRNA targets, which are regulated by AMP1 at the level of translational repression.

Members of the CYP78 family have been shown to control organ size by promoting the length of the cell division phase [29, 32]. Loss of CYP78A function results in smaller leaves whereas enhanced CYP78A expression in leaves increases their growth rate. For CYP78A5 it has been shown that it mediates this effect in a non-cell autonomous manner. Since this function is coupled to the control of plastochron length it was postulated that the factor is part of a compensation mechanism that balances the size of single leaves with the total number of leaves formed [24, 34]. Our work further supports such a function by showing that CYP78A5 overexpression leads to a reduction of leaf number. *amp1* plants also generate more but smaller leaves whereas ubiquitous overexpression of AMP1 did not produce a significant plastochron or leaf size phenotype in our hands. However, recently it was reported that AMP1 overexpressors have an increased rosette size but leaf formation rate was not quantified in this study [43]. Thus, more comprehensive studies including tissue specific overexpression of AMP1 combined with detailed leaf growth analysis are required to assess its role in organ size control and its interaction with CYP78A5 in this process.

## Methods

### Plant materials and growth conditions

*amp1-1* (Col-0; N8324); *amp1-13* (Col-0; N522988), *pt* (Ler; N235), *lamp1-2* (Col-0; N110755), *lamp1-4* (Col-0; N617012), *cyp78a5* (Col-0; N125856) and *cyp78a7* (Col-0; N57134) were ordered from the Nottingham Arabidopsis Stock Centre (http://www.arabidopsis.info/). *pAMP1*::*AMP1-GFP* and *35S*::*AMP1* [12], *pKLU*::*GUS* and *pKLU*::*YFP* [29], *pCLV3*::*GUS* and *pWUS*::*GUS* [44], *pRAP2*.*6L*::*GUS* [22], *35S*::*LAMP1*:*YFP* [10] and *35S*::*PHV*:*YFP* [16] transgenic lines have been described. *35S*::*AMP1* was kindly provided by Thomas Berleth (University of Toronto, Canada) and was generated by fusion of the genomic *AMP1* sequence to the 35S promoter. The T-DNA insertion sites of *cyp78a5* and *cyp78a7* were described previously [34] and both mutations lead to a drastic decrease in the transcript levels of the corresponding genes (S6 Fig). Combinations of mutants and reporter lines were generated by crossing individual lines and homozygous progeny was verified phenotypically and by PCR genotyping.

Arabidopsis seeds were surface sterilized with 70% ethanol containing 0.05% SDS for 3 min, with 96% ethanol for 1 min and dried in a laminar flow hood. After stratification (48h at 4°C) plants were grown *in vitro* on half-strength MS medium (Duchefa) supplemented with 1% sucrose and 0.7% (w/v) agar (Duchefa) or on soil (Platzer) in a growth incubator (BrightBoy GroBank, Model BB-XXL, CLF Plant Climatics) set to long day condition (21°C; 16 h of 80 μmol s^-1^ m^-2^ light/ 8 h dark).

### Gene constructs and transformation

PCR was performed with proofreading thermostable polymerase (Thermo Fisher Scientific), and all clones were confirmed by sequencing. For generation of the plants overexpressing 35S::*CYP785* and 35S::*CYP785*:*MYC* the coding sequence of the gene (AT1G13710) was amplified with primers 5’-CCCGATATCCAGCCTGAG-3’ and 5’-CAAGGAATGTTGGTTTCGCTAGCGGCCGCGGG-3’ and inserted as EcoRV/NotI fragments into corresponding cloning sites of the binary plant expression vector pGWR8 [45] downstream from the constitutive 35S promoter of cauliflower mosaic virus (CaMV). A Myc-tag was subsequently inserted in-frame as a NotI/NotI fragment. To generate plants overexpressing of *35S*::*CYP7875* in pART27 vector we cut *CYP78A5* with KpnI and SalI out from a pH-TOP vector carrying *CYP78A5* and ligated it into KpnI/XhoI sites of pART7 primary cloning vector [46] downstream from the 35S promoter of CaMV. The entire expression cartridge with *CYP5* cDNA (35S-CYP78A5-ocs 3’) was digested with NotI from pART7 and introduced into a NotI cloning site of the pART27 binary vector. To generate *pER>>AMP1*:*MYC*, the coding sequence of AMP1 (AT3G54720) including a C-terminal 6xMYC tag was amplified with primers 5’-GGGCTCGAGATGTCACAACCTCTCACCACCAG-3’ and 5’-TTTACTAGTCTCTAGCGGCCGCCTGTC-3’ cut with XhoI/SpeI and inserted into the corresponding cloning sites of the pER8 estradiol-inducible plant transformation vector [47]. Plants were transformed using the floral dip method, at least 10 independent transformants were generated for each line and the resulting T2 lines were confirmed for single transgene insertion sites based on the 3:1 segregation of the selection marker and propagated for further analysis.

### Fluorescence microscopy

CLSM images of *pKLU*::*YFP* and *pAMP1*::*AMP1-GFP* embryos and seedlings were generated using a TCS SP8 (Leica Microsystems) and a FV1000 (Olympus) confocal laser-scanning microscope. For imaging, we used the following excitation conditions: 488 nm (GFP) and 514 nm (YFP). Confocal laser-scanning microscope settings were kept constant in individual sets of experiments to allow for comparison of reporter proteins.

### RNA *in situ* hybridization

To generate AMP1 and GFP antisense and sense probes, the respective ORFs were amplified from cDNA using corresponding oligos (AMP1probeF: GCTCTCTTATCTTATCCTACGCACA/ AMP1probeR: CGAAGAGACAAAGGCAAAGATGG and eGFPprobeF: ACGGCGTGCAGTGCTTCAG/ eGFPprobeR: TGATCCCGGCGGCGGTCAC), subcloned into pGEM-T Easy, and used as a template for transcription from the T7 or SP6 promoters. 10-d-old Arabidopsis seedlings were fixed with Formaldehyde/Acetic Acid (FAA). The following paraffin infiltration was performed with a Leica ASP200 S tissue processor. Sample preparations and *in situ* hybridizations of 7-mm sections were performed essentially as described [48], a detailed protocol is available online (http://plantdev.bio.wzw.tum.de). Whole mount *in situ* hybridization of embryos and seedlings was performed essentially as described [49]. Images were taken using a BX61 upright microscope (Olympus) with DIC optics.

### Leaf number analysis

The sequential appearance of the leaves was recorded in all experiments by visual observation of plants using a SZX10 stereomicroscope (Olympus) equipped with a DP26 digital camera (Olympus). Cotyledons were considered as leaves. For 10 days old seedlings, a leaf was considered as initiated when its primordium could be observed under the stereomicroscope with 2x magnification. The rosette leaves of 63 days old plants were dissected from the stem and counted.

### Phenotypic analysis of embryos

Whole-mount preparations were done as described [50]. In brief, ovules from siliques of appropriate stages were fixed for 1–4 hours in ethanol/acetic acid (6:1) at room temperature. After washing in 100% ethanol and 70% ethanol, embryos were mounted in a mixture of chloralhydrate/glycerol/water (8:1:2) and cleared for about 1 hour at room temperature. Images were taken with an OLYMPUS BX61 microscope with differential interference contrast (DIC) optics.

### Scanning electron microscopy

Freshly collected seedlings were incubated in FAA fixative (50% ethanol, 10% acetic acid, 5% formaldehyde) overnight at 4°C, dehydrated through a graded ethanol series and subsequently subjected to supercritical point dried using an EM CPD300 (Leica). Shoots were mounted on conductive adhesive tabs (PLANO) and leaf primordia were partially dissected to disclose the shoot meristem. Pictures of samples were taken with a T-3000 tabletop scanning electron microscope (Hitachi). The SEM images were used to measure the SAM surface area with ImageJ software.

### GUS staining

Seedlings were submerged into freshly made GUS staining buffer (100 mM sodium phosphate buffer, 10 mM EDTA, 0.5 mM K_3_Fe(CN)_6_, 0.5 mM K_4_Fe(CN)_6_, Triton X-100 (0.1% v/v) and 1 mM 5-bromo-4-chloro-3-indolyl-β-D-glucuronide). The seedlings were stained at 37°C for various periods of time depending on the reporter strength. After staining the tissue was dehydrated with 70% ethanol. Samples were analyzed using a stereomicroscope (SZX10; Olympus) equipped with a digital camera (DP26; Olympus).

### Microarray experiment

The experiment was conducted with 10 d-old whole seedlings, grown in 96-well plates in liquid MS medium with DMSO in long day conditions. RNA for microarray analysis was extracted from three biological replicates for each line using the RNeasy Kit plant mini kit (Qiagen). cDNA synthesis, labeling and hybridization on a GeneChip Arabidopsis ATH1 genome was performed at the NASC Affymetrix service, University of Nottingham according to standard Affymetrix protocols. The raw microarray data from Affymetrix were uploaded into ArrayStar software (DNASTAR Inc.), preprocessed and normalized using RMA (Robust Microarray Analysis—Quantile) method; statistical significance was assessed with Moderated t-test [51]; p-value was adjusted with FDR (Benjamini and Hochberg false discovery rate ≤0.05) [52]. To reduce the number of false positives and to increase the chances of identifying all the differentially expressed genes, all the genes with adjusted p-values less than 0.05 were selected for further analysis. Genes resulting from this analysis were then filtered with regard to their differential expression (2-fold induction or repression, 95% confidence). The gene ontology term analysis was done using AgriGO web-based tool [53]. The *amp1-13* dataset was already used to analyze the effect of the small molecule hyperphyllin on trancriptomic responses [16]. The microarray data set is available in NCBI’s Gene Expression Omnibus (National Center for Biotechnology Information; http://www.ncbi.nlm.nih.gov)

### RNA and protein analyses

Total RNA was isolated from the whole seedlings using the RNeasy plant mini kit (Qiagen) following the manufacturer’s instructions. The cDNA was synthesized using RevertAid First Strand cDNA Synthesis Kit (Thermoscientific). Gene-specific primers were designed with Primer3 software [54]. Primers are listed in S1 Table.

Plant material (50–100 mg) was flash-frozen in liquid nitrogen and homogenized with a Retsch mill (Verder Scientific). A quantity of 200 μL extraction buffer (100 mM TRIS pH 6.8, 200 mM DTT, 4% SDS, 20% glycerol, 0.2% bromophenol blue) was added and samples were incubated at 95°C for 2 min. The samples were centrifuged at 14,000g for 5 min and 15 μL of the supernatants were separated by SDS-PAGE (10% gel) and semi-dry-blotted onto a polyvinylidene difluoride membrane (Millipore). The membrane was blocked with blocking buffer (5% skim milk powder dissolved in 0.05% Tween 20, 150 mM NaCl, and 10 mM TRIS/HCl, pH 8.0). For CYP78A5-MYC and AMP1:MYC detection membranes were probed with a mouse anti-c-Myc antibody (1:5000, Santa Cruz Biotech). Alkaline phosphatase-conjugated goat anti-mouse IgG (Sigma) diluted 1:5000 with blocking buffer was employed as secondary antibody. For detection the CDP-Star detection reagent (GE Healthcare) was used. For PHV-YFP detection, the membrane was probed with a mouse anti-GFP-HRP antibody (1:10000; # 130-091-833, Miltenyi Biotec) and signals were detected using the ECL Select Detection Reagent (GE Healthcare). For AGO1 detection, membranes were probed with a rabbit anti-AGO 1 antibody (1:10000; AS09527, Agrisera). HRP-goat anti-rabbit IgG (#31460, Invitrogen) diluted 1:10000 with blocking buffer was employed as a secondary antibody. Signals were detected using the ECL Select Detection Reagent (GE Healthcare).

For internal control detection, the detected membrane was incubated in stripping buffer (100 mM 2-mercaptoethanol, 2% SDS, 62.5 mM Tris-HCl pH 6.7) at 50°C for 30 min, and re-probed with monoclonal Anti-Actin antibody produced in mouse (1:10000; A0480, Sigma-Aldrich). HRP-Rabbit Anti-Mouse IgG (61–6020, Invitrogen), diluted 1:5000, was employed as a secondary antibody. Signals were detected using the ECL Select Detection Reagent (GE Healthcare).

### Statistics

Statistical analysis was performed with PRISM8 software (GraphPad Software, San Diego, USA). For statistical analysis of the overlap of misregulated genes we used the Nemates microarray analysis tool (http://nemates.org/MA/progs/overlap_stats.html). Raw data for figures can be found in S3 Table.

## Supporting information

S1 Fig Comparative expression analysis of the 6 *CYP78A* members, *AMP1* and *LAMP1* in the shoot meristem. **(A)** Scheme showing the different expression domains in the shoot meristem used in this analysis (According to: http://arabidopsis.org). **(B)** Relative expression levels of indicated genes in the different shoot meristem domains based on eFB Browser-provided RNAseq expressen data (http://bar.utoronto.ca).
(TIFF)

S2 Fig RAP2.6L expression is upregulated in *cyp78a5,7*. *pRAP2*.*6L*::*GUS* and *pRAP2*.*6L*::*RAP2*.*6L-GUS* activity in wild type and *cyp78a5*,*7* seedlings at 8 DAG. Size bars represent 2 mm.
(TIFF)

S3 Fig LAMP1 overexpression rescues *amp1*-related shoot phenotypes. (**A**) Seedling shoot phenotypes of indicated genotypes at 15 DAG. (**B**) Adult shoot phenotypes of indicated genotypes at 32 DAG. (**C**) Quantification of rosette leaf number in the indicated genotypes at 7 DAG, 9 DAG and 15 DAG (means ± SE of the mean; n ≥ 10). Different letters over the error bars indicate significant differences within day-specific graphs (P < 0.05; one-way ANOVA followed by Tukey’s multiple comparison tests). Size bars represent 2 mm (A) and 2 cm (B).
(TIFF)

S4 Fig Overexpression of *CYP785* affects leaf number and SAM size. **(A)** Seedling shoot phenotype of wild type, *35S*::*CYP78A5* and *35S*::*CYP78A5*:*MYC* at 10 DAG (upper panel) and at 14 DAG (lower panel). **(B)** Scanning electron micrographs of shoot apices from 10-d-old plants of indicated genotypes. **(C)** Quantification of rosette leaf number in indicated genotypes at 10 DAG and at 14 DAG (means ± SE of the mean; n ≥ 10). **(D)** SAM size measurement of the indicated genotypes at 10 DAG (means ± SE of the mean; n ≥ 5). **(E)** Immunoblotting of protein extracts of 10-d-old wild type and *35S*::*CYP78A5*:*MYC* seedlings. CYP78A5:MYC was detected using an anti-MYC antibody. Size bars represent 1mm (A) and 500 μm (B).
(TIFF)

S5 Fig AGO1 and PHV-YFP protein levels are increased in *cyp78a5,7*. **(A)** Immunoblotting of protein extracts of 10-d-old *35S*::*PHV-YFP* seedlings in the indicated genetic backgrounds. Results of three independent biological repeats (BR) are shown. Upper panel: PHV-YFP detection using an anti-GFP antibody. Lower panel: Actin detection using an anti-actin antibody served as a loading control. **(B)** Immunoblotting of protein extracts from the indicated genotypes. Results of three independent biological repeats (BR) are shown. Upper panel: AGO1 detection using an anti-AGO1 antibody. Lower panel: Actin detection using an anti-actin antibody served as a loading control. **(C)** Quantification of relative signal intensities of PHV-YFP bands shown in (A) normalized against corresponding actin band intensities in wild type and *cyp78a5*,*7* (means ± SE of the mean; n = 3). ** indicates a significant difference (Student’s 2-tailed t-test; p < 0.01). **(D)** Quantification of relative signal intensities of AGO1 bands shown in (B) normalized against corresponding actin band intensities in wild type and *cyp78a5*,*7* (means ± SE of the mean; n = 3). ** indicates a significant difference (Student’s 2-tailed t-test; p < 0.01).
(TIFF)

S6 Fig Expression analysis of *CYP78A5* and *CYP78A7* in *cyp78a5* and *cyp78a7*. **(A)** Detection of *CYP78A5* transcript levels in 7-d-old seedlings of the indicated genotypes by semiquantitative RT-PCR. In *cyp78a5* no CYP78A5-specific cDNA fragment (~970 bp) could be detected. **(B)** Detection of *CYP78A7* transcript levels in 7-d-old seedlings of the indicated genotypes by semiquantitative RT-PCR. In *cyp78a7* no CYP78A7-specific cDNA fragment (~1100 bp) could be detected. *UBQ5* (~250 bp) was used as normalization control.
(TIFF)

S1 Table List of genes differently expressed in *cyp78a5,7* and *amp1-13* seedlings. (XLSX)

S2 Table List of primers used in this study. (DOCX)

S3 Table Raw data for figures. (XLSX)
