# Supplementary figures and images for: AMP1 and CYP78A5/7 act through a common pathway to govern cell fate maintenance in Arabidopsis thaliana
Source: PLoS Genet. 2020 Sep 22;16(9):e1009043. doi: 10.1371/journal.pgen.1009043 (PMC7531801; doi:10.1371/journal.pgen.1009043)

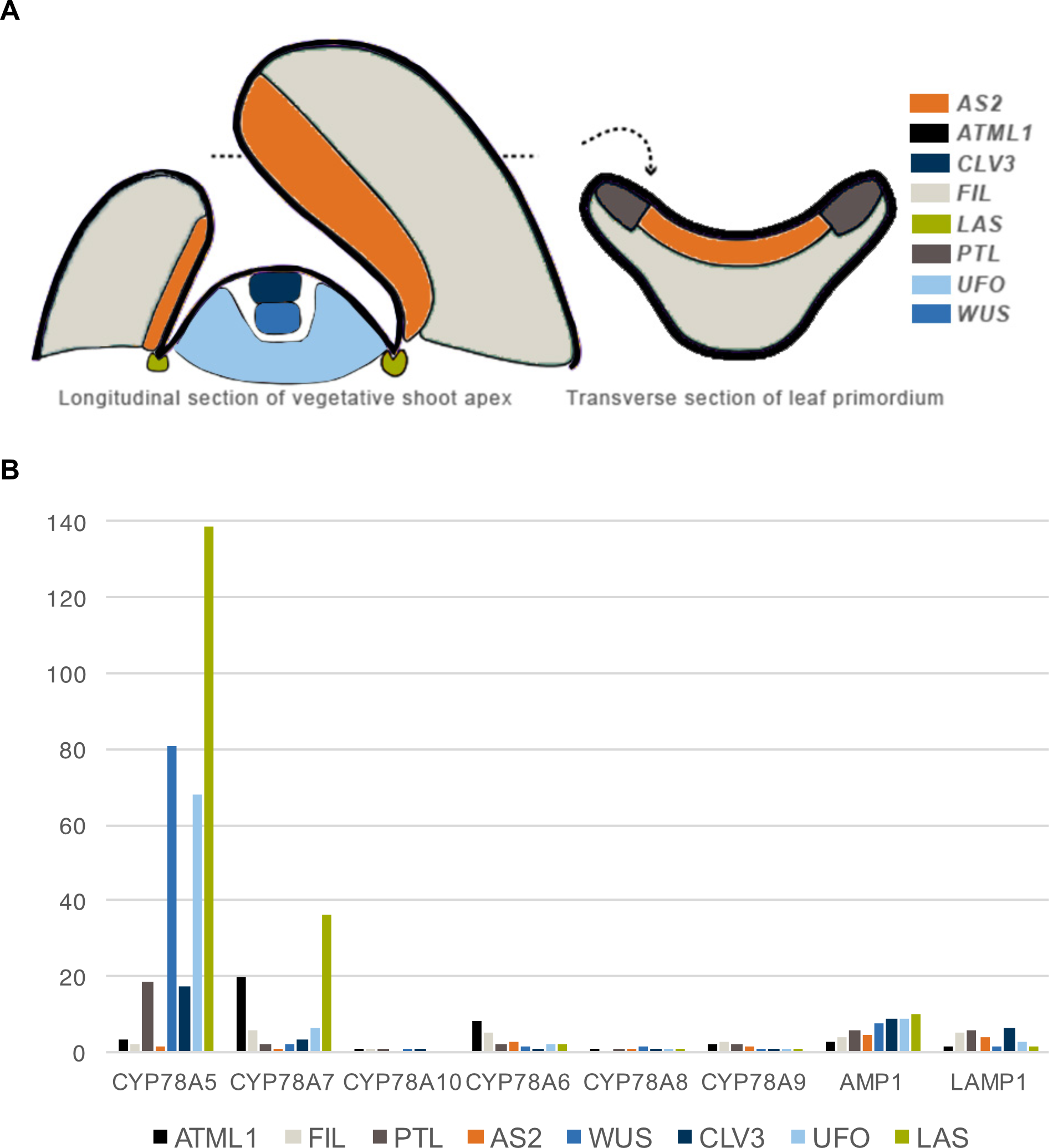

Supplement: S1 Fig — (A) Scheme showing the different expression domains in the shoot meristem used in this analysis (According to: http://arabidopsis.org). (B) Relative expression levels of indicated genes in the different shoot meristem domains based on eFB Browser-provided RNAseq expressen data (http://bar.utoronto.ca). (TIFF) [file pgen.1009043.s001.tiff]

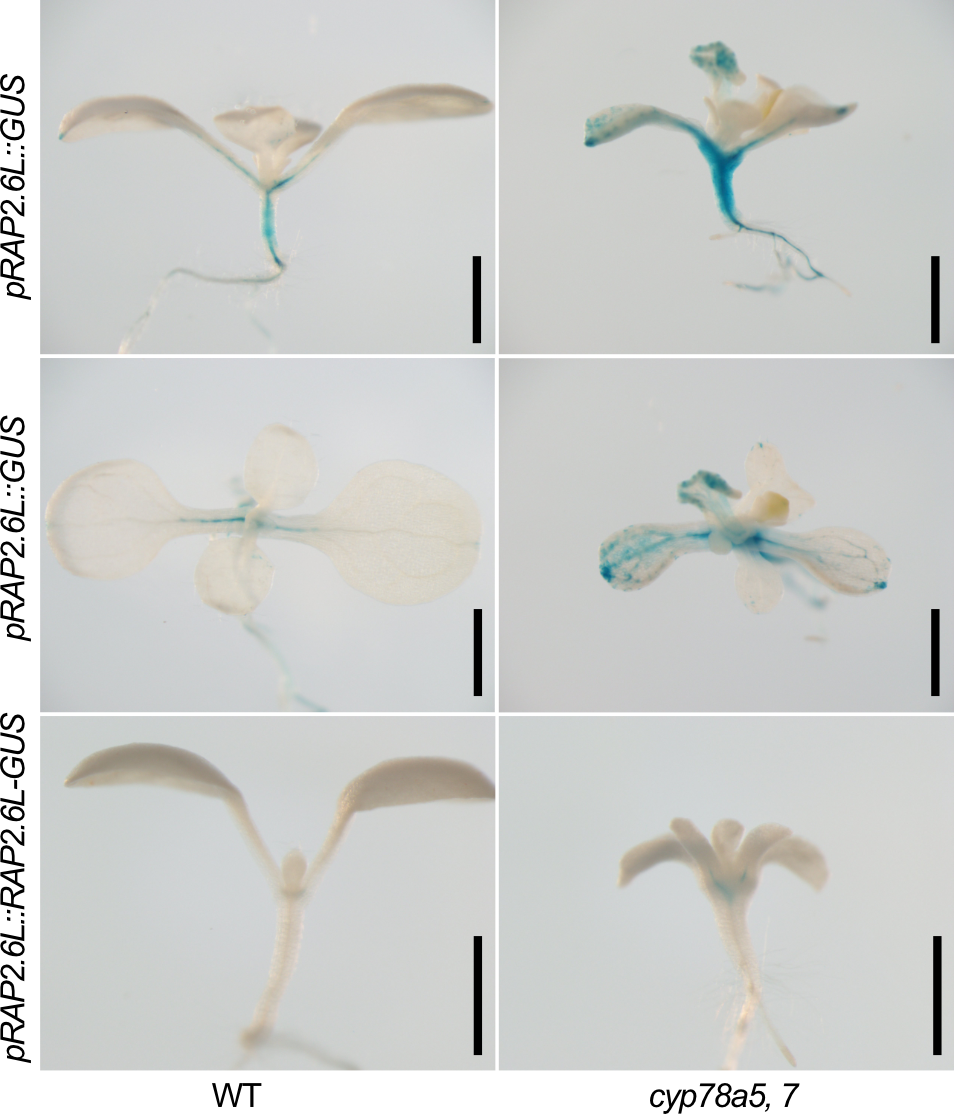

Supplement: S2 Fig — pRAP2.6L::GUS and pRAP2.6L::RAP2.6L-GUS activity in wild type and cyp78a5,7 seedlings at 8 DAG. Size bars represent 2 mm. (TIFF) [file pgen.1009043.s002.tiff]

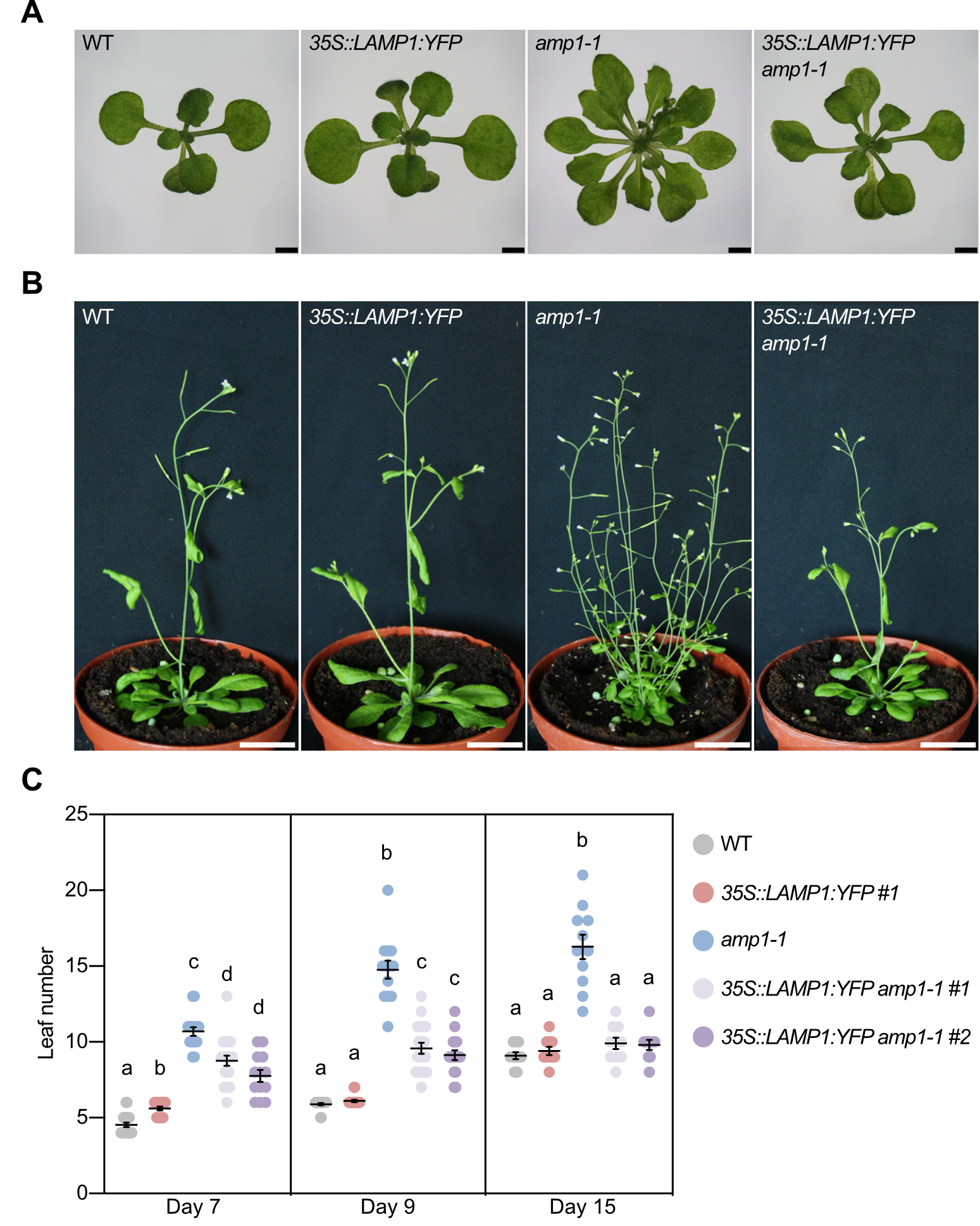

Supplement: S3 Fig — (A) Seedling shoot phenotypes of indicated genotypes at 15 DAG. (B) Adult shoot phenotypes of indicated genotypes at 32 DAG. (C) Quantification of rosette leaf number in the indicated genotypes at 7 DAG, 9 DAG and 15 DAG (means ± SE of the mean; n ≥ 10). Different letters over the error bars indicate significant differences within day-specific graphs (P < 0.05; one-way ANOVA followed by Tukey’s multiple comparison tests). Size bars represent 2 mm (A) and 2 cm (B). (TIFF) [file pgen.1009043.s003.tiff]

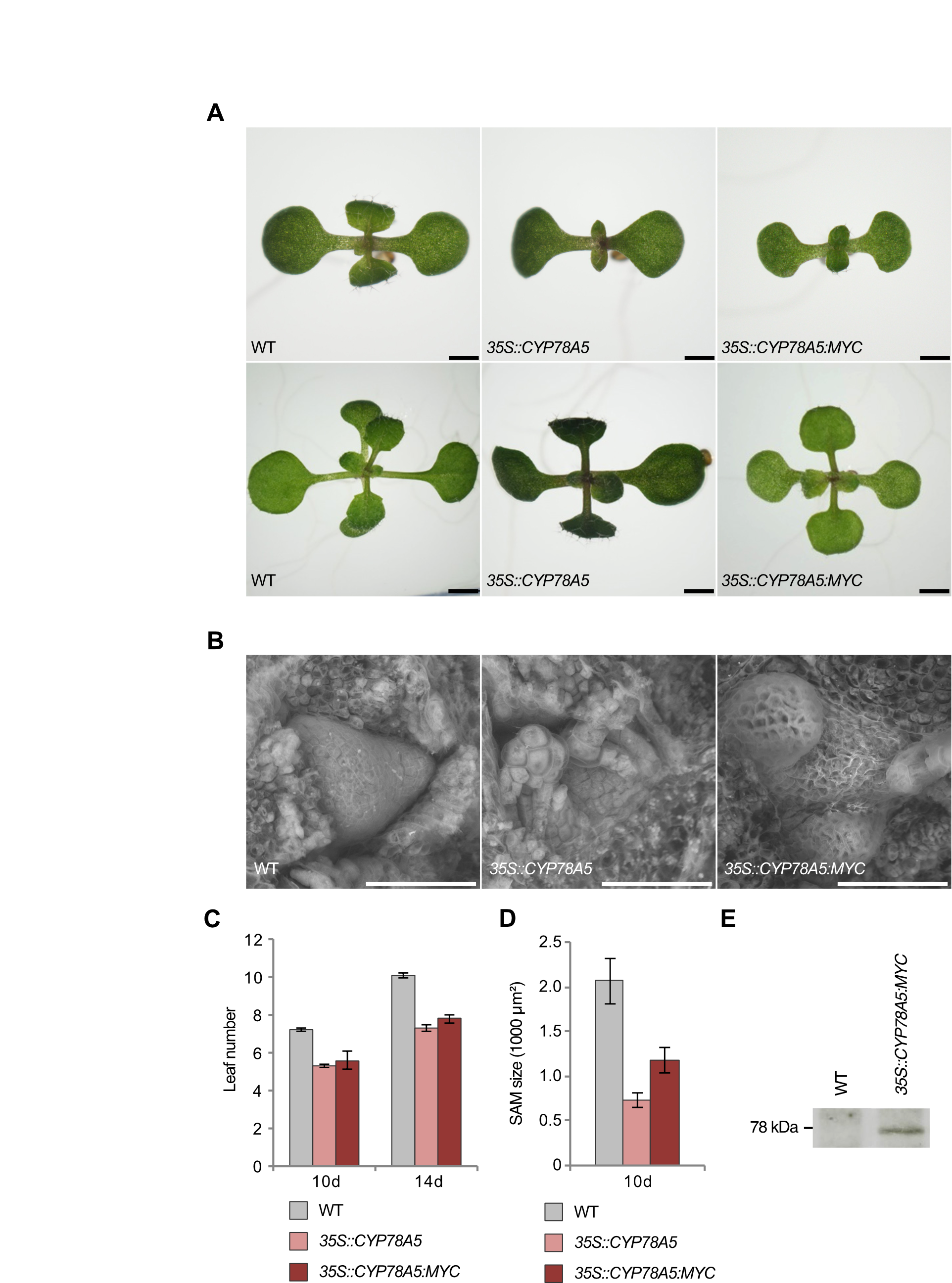

Supplement: S4 Fig — (A) Seedling shoot phenotype of wild type, 35S::CYP78A5 and 35S::CYP78A5:MYC at 10 DAG (upper panel) and at 14 DAG (lower panel). (B) Scanning electron micrographs of shoot apices from 10-d-old plants of indicated genotypes. (C) Quantification of rosette leaf number in indicated genotypes at 10 DAG and at 14 DAG (means ± SE of the mean; n ≥ 10). (D) SAM size measurement of the indicated genotypes at 10 DAG (means ± SE of the mean; n ≥ 5). (E) Immunoblotting of protein extracts of 10-d-old wild type and 35S::CYP78A5:MYC seedlings. CYP78A5:MYC was detected using an anti-MYC antibody. Size bars represent 1mm (A) and 500 μm (B). (TIFF) [file pgen.1009043.s004.tiff]

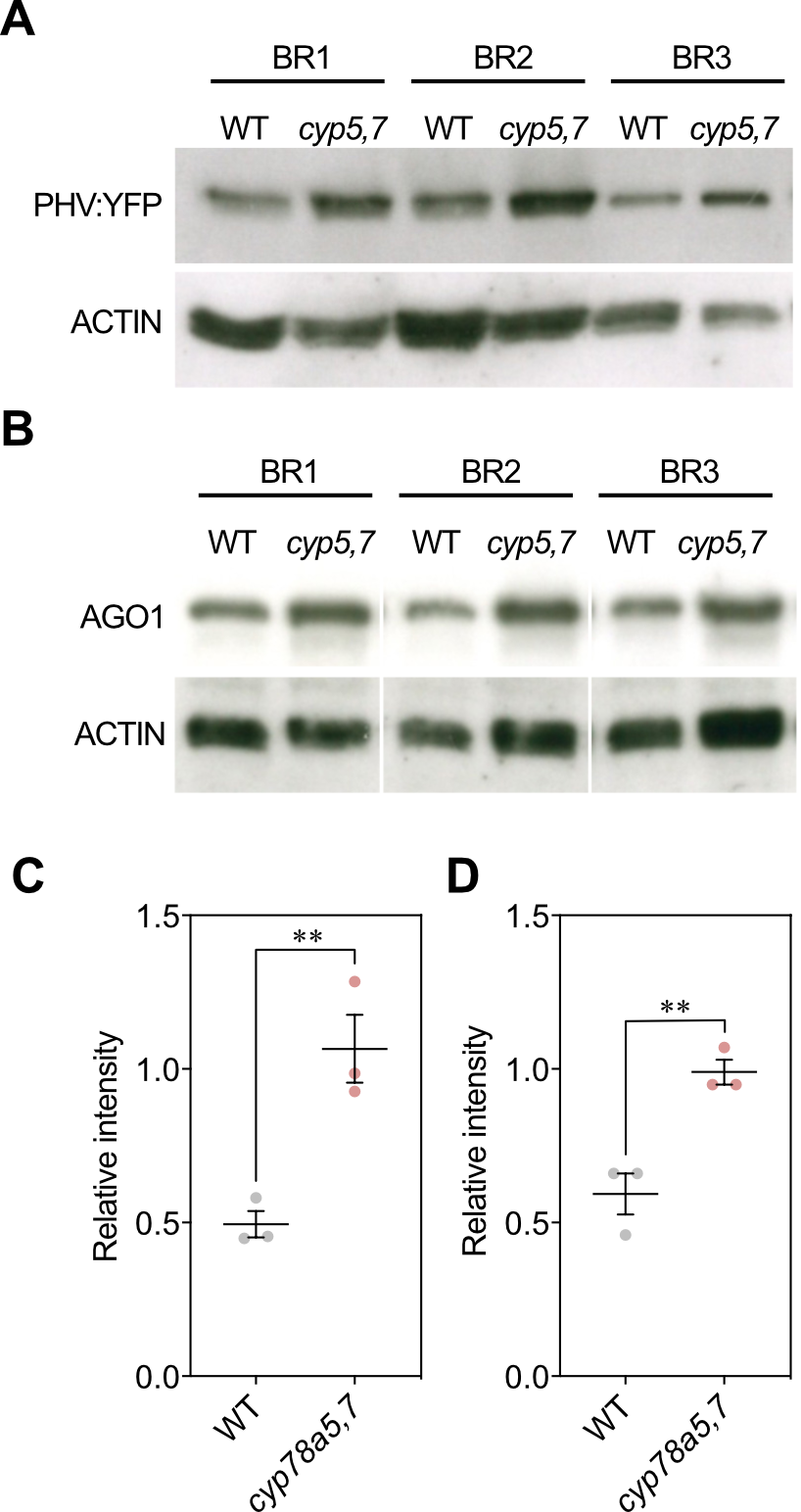

Supplement: S5 Fig — (A) Immunoblotting of protein extracts of 10-d-old 35S::PHV-YFP seedlings in the indicated genetic backgrounds. Results of three independent biological repeats (BR) are shown. Upper panel: PHV-YFP detection using an anti-GFP antibody. Lower panel: Actin detection using an anti-actin antibody served as a loading control. (B) Immunoblotting of protein extracts from the indicated genotypes. Results of three independent biological repeats (BR) are shown. Upper panel: AGO1 detection using an anti-AGO1 antibody. Lower panel: Actin detection using an anti-actin antibody served as a loading control. (C) Quantification of relative signal intensities of PHV-YFP bands shown in (A) normalized against corresponding actin band intensities in wild type and cyp78a5,7 (means ± SE of the mean; n = 3). ** indicates a significant difference (Student’s 2-tailed t-test; p < 0.01). (D) Quantification of relative signal intensities of AGO1 bands shown in (B) normalized against corresponding actin band intensities in wild type and cyp78a5,7 (means ± SE of the mean; n = 3). ** indicates a significant difference (Student’s 2-tailed t-test; p < 0.01). (TIFF) [file pgen.1009043.s005.tiff]

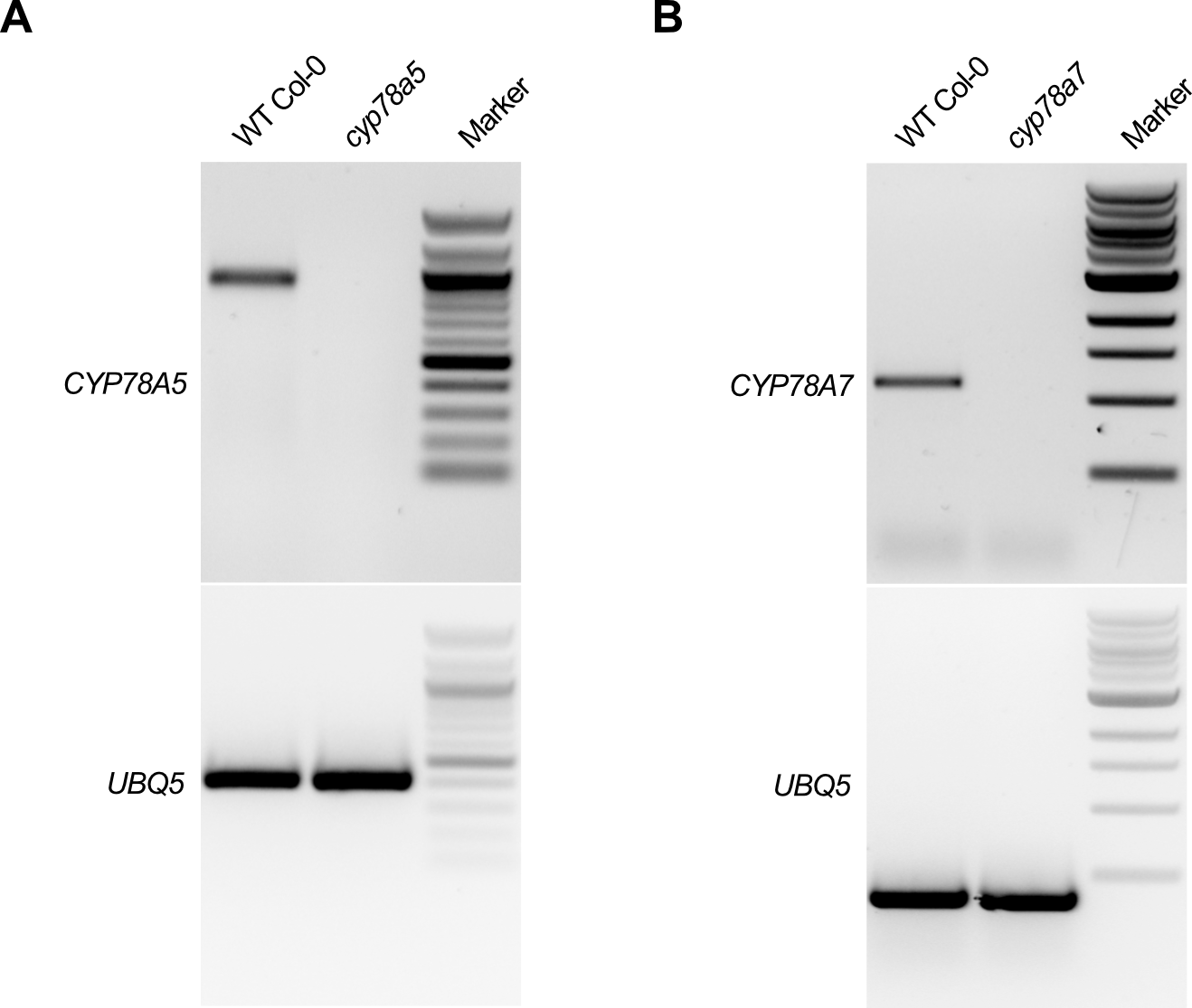

Supplement: S6 Fig — (A) Detection of CYP78A5 transcript levels in 7-d-old seedlings of the indicated genotypes by semiquantitative RT-PCR. In cyp78a5 no CYP78A5-specific cDNA fragment (~970 bp) could be detected. (B) Detection of CYP78A7 transcript levels in 7-d-old seedlings of the indicated genotypes by semiquantitative RT-PCR. In cyp78a7 no CYP78A7-specific cDNA fragment (~1100 bp) could be detected. UBQ5 (~250 bp) was used as normalization control. (TIFF) [file pgen.1009043.s006.tiff]
