# Supplementary material for: AMP1 and CYP78A5/7 act through a common pathway to govern cell fate maintenance in Arabidopsis thaliana
Source: PLoS Genet. 2020 Sep 22;16(9):e1009043. doi: 10.1371/journal.pgen.1009043 (PMC7531801; doi:10.1371/journal.pgen.1009043)
Supplement: S2 Table — (DOCX) [file pgen.1009043.s008.docx]

| Name | Sequence 5’-3’ | Purpose |
| --- | --- | --- |
| AMP1F1 | TATCAGTGGCTGGAATTTGG | PCR-genotyping |
| amp1-1F1 | TATCAGTGGCTGGAATTTGA | PCR-genotyping |
| AMP1R | GCTCTCTGAATCGCTCTTGC | PCR-genotyping |
| AMP1cDNAF (XhoI) | GGGCTCGAGATGTCACAACCTCTCACCACCAG | Cloning |
| EST>>MYC R1 (SpeI) | TTTACTAGTCTCTAGCGGCCGCCTGTC | Cloning |
| LAMP1 F3 | AGCAAACAACCAACTCCATTG | PCR-genotyping |
| LAMP1 F4 | AGTCGTTGGATCACCTAACCC | PCR-genotyping |
| LAMP1 R3 | TAACAGTTTCCCCCTGAAACC | PCR-genotyping |
| LAMP1 R4 | TGGTAAAAGCTGACAAATTAATGTTC | PCR-genotyping |
| DSPM | TACGAATAAGAGCGTCCATTTTAGAGTGA | PCR-genotyping |
| LBb1.3 | ATTTTGCCGATTTCGGAAC | PCR-genotyping |
| KLUFgenotype | CACTCTCTCTCTCCTGCCATAAC | PCR-genotyping and RT-PCR |
| KLURgenotype | CATCCTTTTGCAAGCCAAGCA | PCR-genotyping and RT-PCR |
| CYP78A7Fgenotype | AAACCGACGTCTTTCTTCCCATGCA | PCR-genotyping and RT-PCR |
| CYP78A7Rgenotype | CGATGATTTTCTTGACGAGGGTTC | PCR-genotyping and RT-PCR |
| CYP78A5 ORF F | AAGCGGCCGCTTTCTCTTGGGTAGTTATAATAAT | Cloning |
| CYP78A5 ORF R | CCCGATATCCAGCCTGAG | Cloning |
| AMP1probeF | GCTCTCTTATCTTATCCTACGCACA | in situ hybridization |
| AMP1probeR | CGAAGAGACAAAGGCAAAGATGG | in situ hybridization |
| eGFPprobeF | ACGGCGTGCAGTGCTTCAG | in situ hybridization |
| eGFPprobeR | TGATCCCGGCGGCGGTCAC | in situ hybridization |
| AMP1RT-F | GGAATTCTCTTGGCTGATGAGC | RT-PCR |
| AMP1RT-R | GTGAAACCTCCTTTAAGAGCTTTGC | RT-PCR |

**S2 Table: List of primers used in this study.**
